# Isolation and X-ray characterization of palladium–N complexes in the guanylation of aromatic amines. Mechanistic implications

**DOI:** 10.3762/bjoc.9.165

**Published:** 2013-07-22

**Authors:** Abdessamad Grirrane, Hermenegildo Garcia, Eleuterio Álvarez

**Affiliations:** 1 Instituto Universitario de Tecnología Química CSIC-UPV, Universidad Politécnica de Valencia, Av. De los Naranjos s/n, 46022 Valencia, Spain; 2 Instituto de Investigaciones Químicas CSIC-US, Departamento de Química Inorgánica, Av. Américo Vespucio 49, 41092 Sevilla, Spain

**Keywords:** coordination chemistry, guanylation reaction, homogeneous catalysis, palladium complexes, reactive intermediates

## Abstract

In the context of palladium-catalyzed guanylation of anilines herein, we have been able to characterize and isolate bis(anilino) and bis(guanidino)Pd(II) complexes using reaction conditions under which stoichiometric amounts of palladium salts are used. Characterization of these palladium complexes strongly supports a mechanistic proposal for the catalytic guanylation of anilines using PdCl_2_(NCCH_3_)_2_ as catalyst that involves the intermediacy of these Pd(II) complexes.

## Introduction

**Scheme 1:**
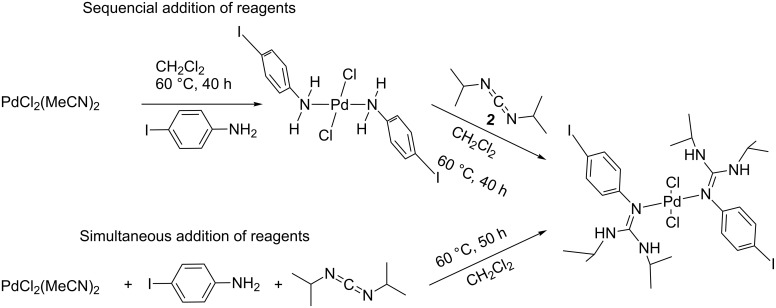
Isolation of *trans*-dichlorobis(4-iodoanilino-*ĸN*)palladium(II) and *trans*-dichlorobis[1,3-diisopropyl-2-(4-iodophenyl)guanidino-*ĸN*(aryl)]palladium(II) complexes formed by reaction of PdCl_2_(MeCN)_2_ with 4-iodoaniline (upper row) or with 4-iodoaniline and *N*,*N*’-diisopropylcarbodiimide (lower reaction).

*N*-Arylguanidines are important compounds with interesting biological activities [1–2] such as fungicides [3] and also in supramolecular chemistry as complementary partners of carboxylate and nitro groups [4–7]. Some of these guanidines are commercially used as antifouling agents in marine paints and in the formulation of protective surface coatings [8–10]. *N*-Arylguanidines can be obtained by aniline insertion into the corresponding carbodiimide [11–18]. This nucleophilic addition can be efficiently catalyzed by palladium salts [19], such as PdCl_2_ or Pd(OAc)_2_ in homogenous phase. Also recently we have reported that palladium nanoparticles supported on magnesia can be a solid catalyst for this process [20]. Working with PdCl_2_(NCCH_3_)_2_ in dichloromethane we were able to isolate two types of palladium complexes with iodoaniline and guanidine, respectively, (see Scheme 1) that give some clue about the reaction mechanism of the catalytic process.

## Results and Discussion

**Scheme 2:**
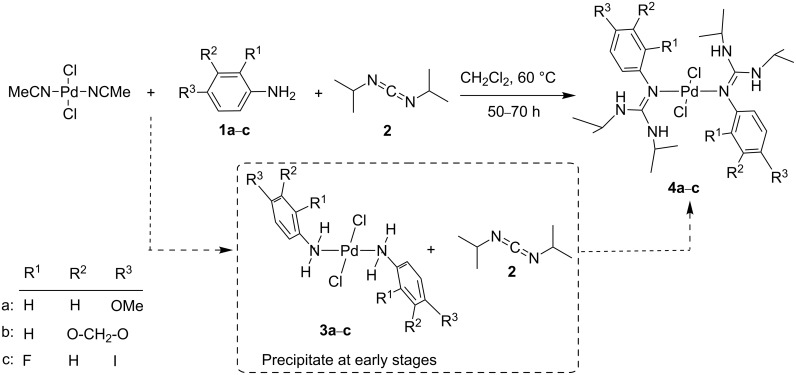
Isolation of *trans*-dichlorobis[1,3-diisopropyl-2-(aryl)guanidino-*ĸN*(aryl)]palladium(II) complexes (**4a**–**c**) by reaction of PdCl_2_(MeCN)_2_ with anilines **1a**–**c** and *N*,*N*’-diisopropylcarbodiimide (**2**). Dashed lines indicate the formation of *trans*-dichlorobis(anilino-*ĸN*)palladium(II) complexes (**3a**–**c**) as primary intermediates during the reaction, and their subsequent reaction with **2** to led **4a**–**c**.

In order to provide further support to the mechanistic proposal for the C–N insertion promoted by palladium(II) suggested by us [20], in the present report we describe the study of palladium-catalyzed guanylation of three additional anilines (**1a**–**c**) with *N*,*N*’-diisopropylcarbodiimide (**2**). For these reactions we have been able to characterize three palladium complexes of the type of bis(anilino)Pd(II) (**3a**–**c**) as well as three palladium complexes of the type of bis(guanidino)Pd(II) (**4a**–**c**) (Scheme 2) whose structures have been characterized by single-crystal X-ray structural analysis, as well as to obtain evidence for the intermediacy of these complexes in the catalytic process. Overall the present data reinforce the previous proposal for the mechanism of aniline guanylation.

When a stoichiometric (2:2:1) mixture of anilines **1a**–**c** and carbodiimide **2** with PdCl_2_(MeCN)_2_ is stirred at 60 °C in CH_2_Cl_2_, evolution at initial reaction times of a solid precipitate is observed (Scheme 2). Filtration of these precipitates and subsequent washing with CH_2_Cl_2_ renders three solids whose combustion analysis is in accordance with the percentages expected for dichlorobis(anilino-*ĸN*)palladium(II) (**3a**–**c**) (see Supporting Information File 1, experimental section). IR spectra of complexes **3a**–**c** show the characteristic absorption peaks due to the coordinated anilines **1a**–**c** (see Supporting Information File 1, Figures S1–S3); these are compatible with the proposed structure for these intermediates. Complex **3a** derived from **1a** has been recently characterized by single-crystal X-ray diffraction [21] showing similar coordination to *trans*-dichlorobis(4-iodoaniline-*ĸN*)palladium(II) complex recently published by us [20]. Compounds **3a**–**c** were also characterized by solid state ^13^C NMR spectroscopy that gave spectra showing carbon peaks compatible with the proposed structure (see Supporting Information File 1, Figures S4–S6).

**Figure 1:**
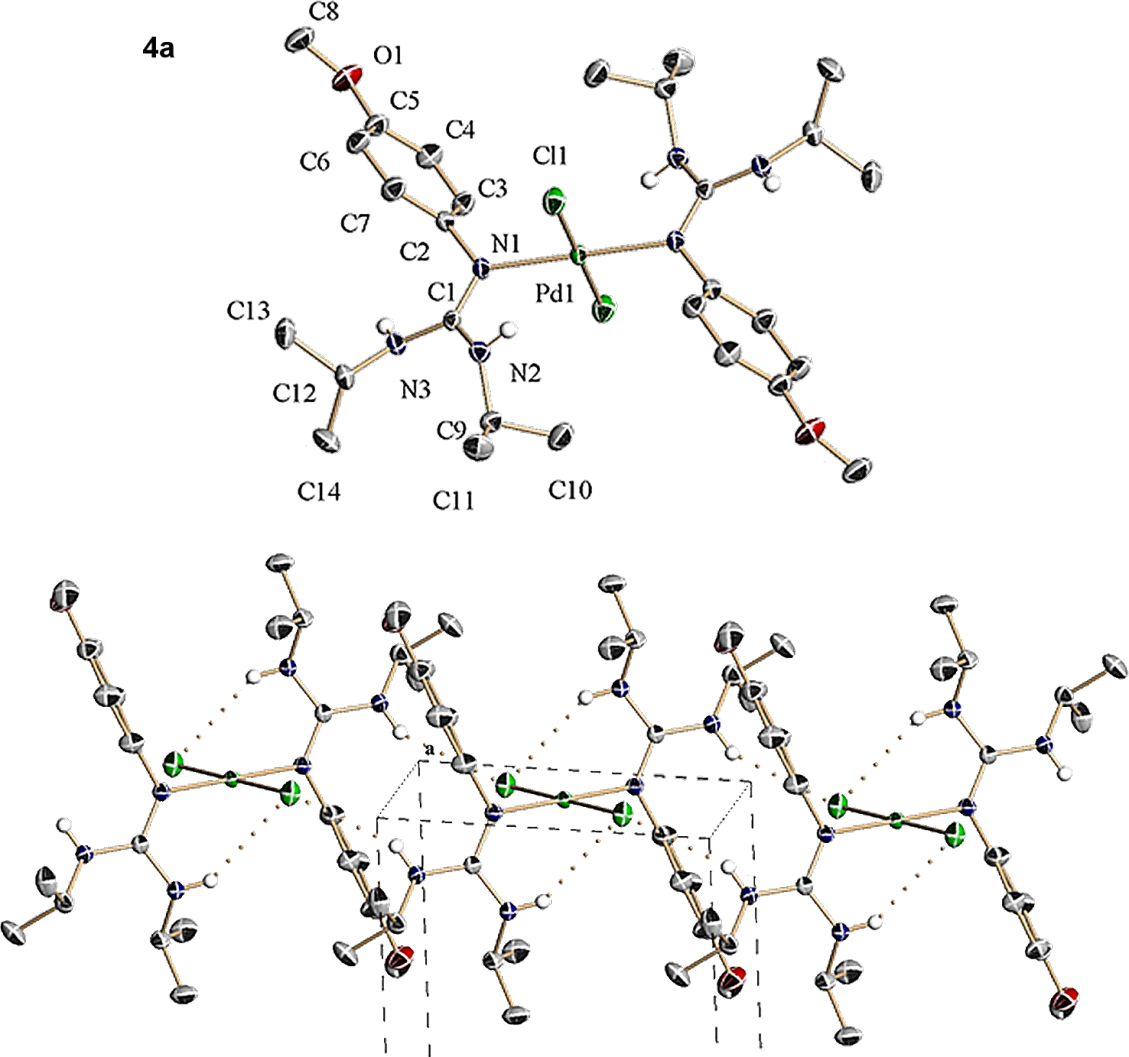
(Top) ORTEP view of the centrosymmetric molecule **4a**. (Bottom) Crystal packing detail of **4a** viewed along the *a*-axis showing the presence of inter- and intramolecular hydrogen bonds between Cl and H (NH groups) atoms.

**Figure 2:**
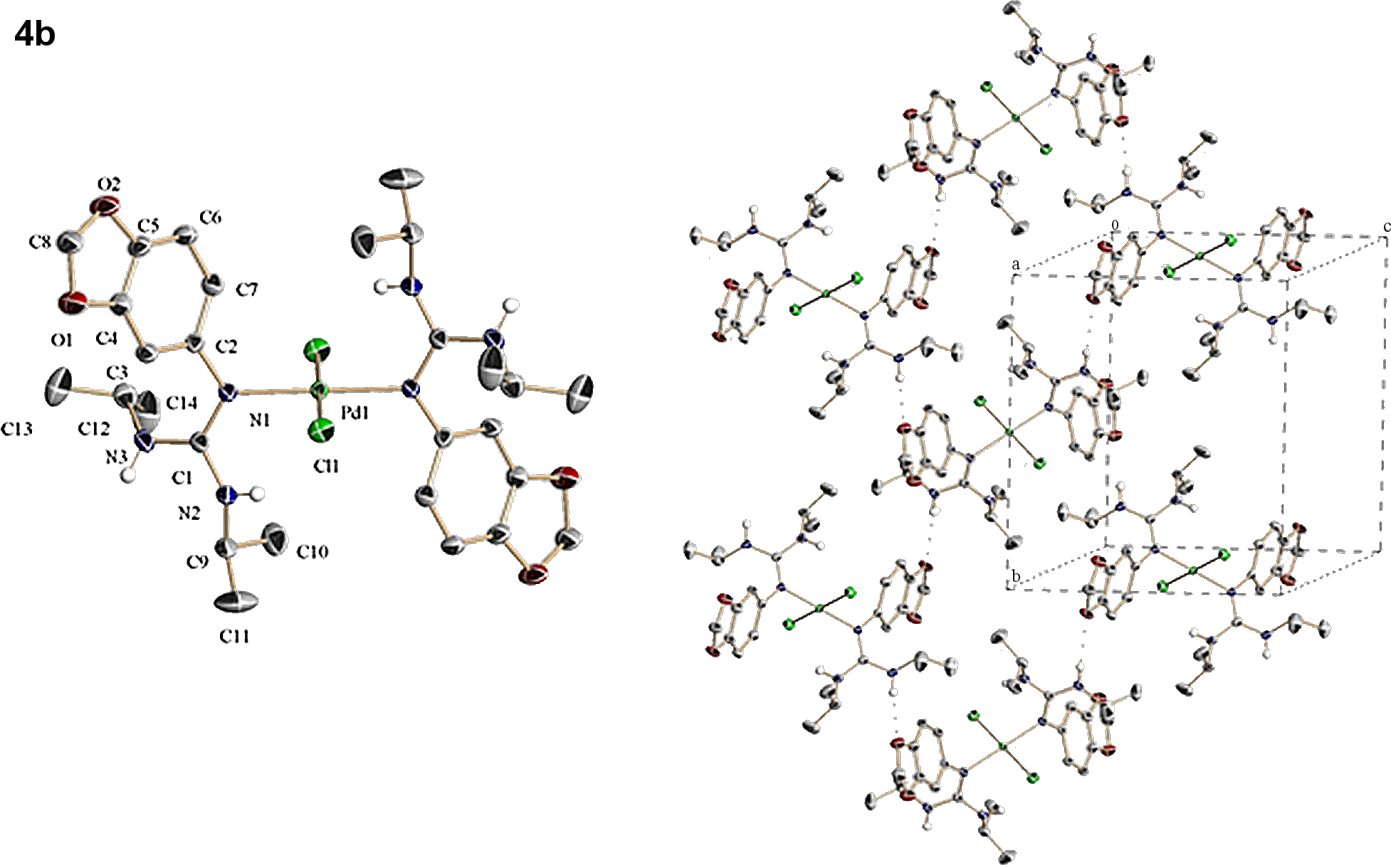
(Left) ORTEP representation of **4b**. (Right) Crystal packing detail of **4b** viewed along the *a*-axis showing the presence of intermolecular hydrogen bonds between O (from the dioxole moieties) and N (NH groups) atoms.

**Figure 3:**
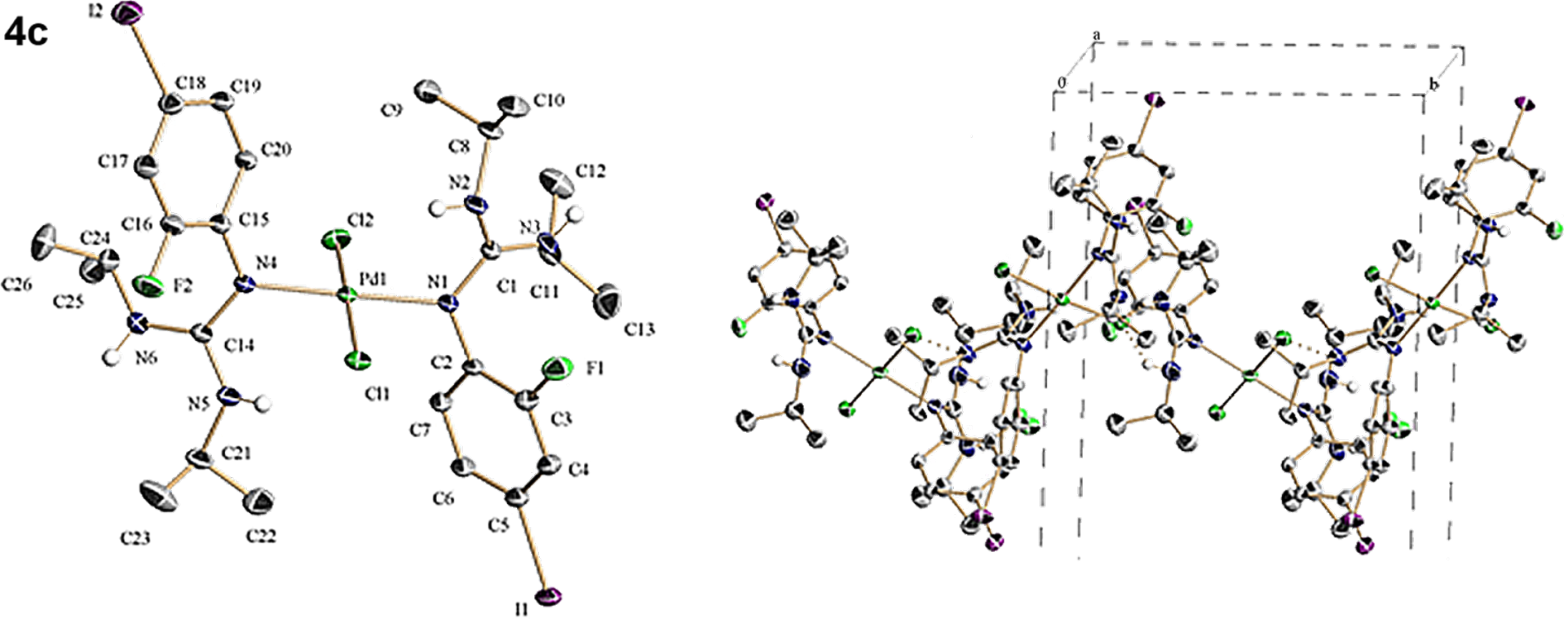
(Left) ORTEP representation of **4c**. (Right) Crystal-packing detail of **4c** viewed along the *a*-axis showing the presence of intermolecular hydrogen bonds between Cl and N (NH groups) atoms.

After prolonging the reaction time, the initially evolved precipitate undergoes dissolution indicating that it has been transformed under the reaction conditions. At this stage, filtering of the transparent orange-red (**4a**,**b**) and red (**4c**) solutions followed by subsequent addition of ethyl ether or toluene and slow solvent evaporation at ambient temperature allows the formation of crystals with suitable quality for a crystallographic diffraction study. The structures of these intermediates solved by X-ray analysis showed that these compounds corresponding to *trans*-dichlorobis[arylguanidino-*ĸN*(aryl)]palladium(II). Figure 1, Figure 2 and Figure 3 present ORTEP views of complexes **4a**–**c** as well as selected views along some crystallographic axes (see Tables S1–S3 and also Figures S7, S11 and S15 in Supporting Information File 1, and for full details of the crystallographic data see Supporting Information File 2).

Besides X-ray crystal structure analysis, palladium complexes **4a**–**c** were also characterized by NMR spectroscopy, ESIMS and combustion analysis (see experimental section in Supporting Information File 1). ^1^H, ^13^C and ^19^F NMR spectroscopy of **4a**–**c** in CD_2_Cl_2_ solution provides evidence showing that under the reaction conditions the starting reagents (PdCl_2_(MeCN)_2_, **1a**–**c** and **2**) including intermediates bis(anilino-*ĸN*)palladium(II) (**3a**–**c**) are completely converted into the corresponding bis(guanidino-*ĸN*)palladium(II) (**4a**–**c**) (see Supporting Information File 1, Figures S8 and S9 for **4a**, Figures S12 and S13 for **4b** and Figures S16–S18 for **4c**). ESIMS of a solution obtained after dissolving complexes **4a**–**c** in CH_2_Cl_2_/CH_3_CN (1:1) shows single positive MS peaks at 639.3, 667.2 and 867.1 attributable, respectively, to the complexes [C_28_H_46_Cl_2_N_6_O_2_Pd (**4a**) − Cl^−^]**^+^**, [C_28_H_42_Cl_2_N_6_O_4_Pd (**4b**) − Cl^−^]^+^ and [C_26_H_38_Cl_2_F_2_I_2_N_6_Pd (**4c**) − Cl^−^]**^+^**. Also negative MS shows single peaks at 711.2, 739.1 and 938.9 attributable, respectively, to the complexes [C_28_H_46_Cl_2_N_6_O_2_Pd (**4a**) + Cl^−^]^−^, [C_28_H_42_Cl_2_N_6_O_4_Pd (**4b**) + Cl^−^]^−^ and [C_26_H_38_Cl_2_F_2_I_2_N_6_Pd (**4c**) + Cl^−^]^−^ (see Supporting Information File 1, Figures S10, S14 and S19).

**Scheme 3:**
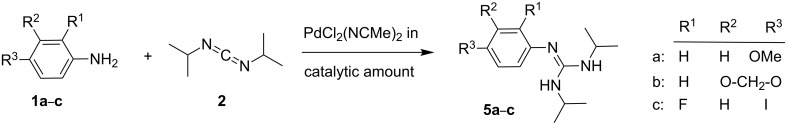
Guanylation reactions of anilines **1a**–**c** by *N*,*N*’-diisopropylcarbodiimide (**2**) catalyzed by Pd(II) salt.

Similar reactions were carried out mixing anilines **1a**–**c** and *N*,*N*’-diisopropylcarbodiimide (**2**), but in this case in the presence of only a catalytic amount of PdCl_2_(NCCH_3_)_2_ (4 mol %) (Scheme 3). Under these conditions no evidence for the formation of palladium complexes (**3a**–**c**) and (**4a**–**c**) could be obtained due to the low amount of palladium and no solid precipitates were observed. In contrast, in the presence of catalytic amounts of palladium, formation of the corresponding *N*-arylguanidines was observed in almost quantitative yield. These guanidines **5a**–**c** formed by nucleophilic attack of anilines **1a**–**c** to *N*,*N*’-diisopropylcarbodiimide (**2**) catalyzed by palladium were fully characterized by analytical and spectroscopic data (see Supporting Information File 1, experimental section).

**Figure 4:**
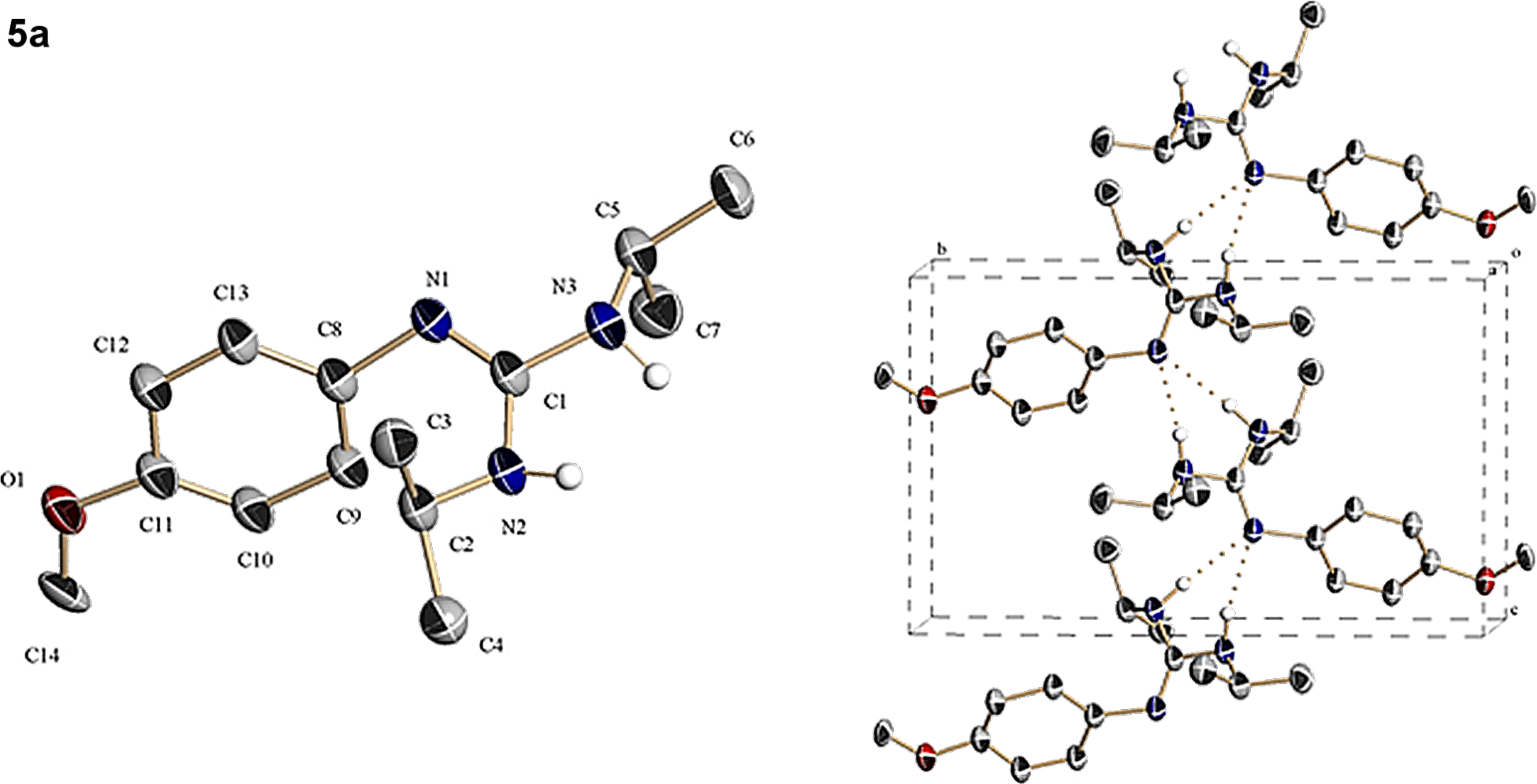
(Left) ORTEP representation of **5a**. (Right) Crystal packing details of **5a** viewed along the *a*-axis showing the presence of intermolecular hydrogen bonds between N atoms.

Structure of guanidine **5a** was confirmed by single-crystal X-ray analysis, Figure 4 shows the corresponding ORTEP for compound **5a** as well as some views of the crystal packing (see also Supporting Information File 1, Table S4 and Figure S20 and for full details of crystallographic data see Supporting Information File 2). Beside X-ray crystal analysis of guanidine **5a**, guanidines **5a–c** were also characterized by ^1^H, ^13^C and ^19^F NMR spectroscopy and combustion analysis (see Figures S21 and S22 for **5a**, Figures S24 and S25 for **5b**, our recent published work for **5c** [20] and experimental section in Supporting Information File 1). ESIMS and GC–MS of solutions obtained respectively after dissolving guanidines **5a** and **5b** in CH_2_Cl_2_/MeOH (1:1) and CH_2_Cl_2_ shows a single positive MS peak at 250.2 and 263.2 Da attributable, respectively, to the complexes [C_14_H_23_N_3_O (**5a**) + H**^+^**]**^+^** and C_14_H_21_N_3_O_2_ (**5b**) (see Figure S23 and Figure S26 in Supporting Information File 1).

**Scheme 4:**
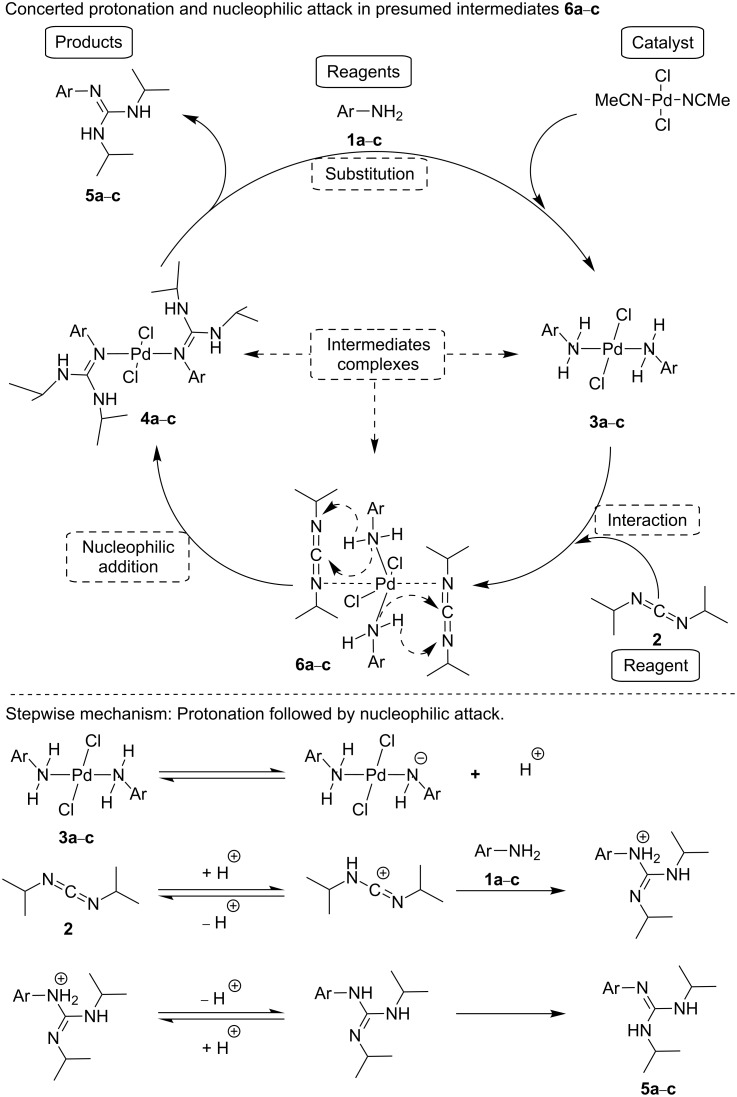
Possible mechanisms for the C–N coupling catalyzed by PdCl_2_(NCMe)_2_ in homogeneous phase.

Overall the information obtained from the experiments performed in the presence of a large palladium excess, in which two kinds of palladium complexes have been detected and isolated, with the formation of guanidines under conditions in which a catalytic amount of palladium is present, allows us to make reasonable mechanistic proposals. Thus, upon contacting anilines **1a**–**c** and PdCl_2_(MeCN)_2_, a rapid formation of dichlorobis(anilino-*ĸN*)palladium(II) (**3a**–**c**) complexes should take place. These palladium complexes will interact with the *N*,*N*’-diisopropylcarbodiimide (**2**) giving rise to the dichlorobis(guanidino-*ĸN*)palladium(II) (**4a**–**c**) complexes. When Pd is used in catalytic amounts, cleavage of this bis(guanidino-*ĸN*) complex by aniline will form another dichlorobis(anilino-*ĸN*)palladium(II) (**3a**–**c**) completing one cycle and liberating guanidines **5a**–**c** as free products of this catalytic reaction with high yields and selectivities (see Scheme 4). In this mechanism the rate determining step will be the attack of dichlorobis(anilino-*ĸN*)palladium(II) (**3a**–**c**) to the *N*,*N*’-diisopropylcarbodiimide (**2**) (Scheme 4).

In this process, coordination of nitrogen to palladium should strongly reduce the nucleophilicity of the corresponding nitrogen atom and, therefore, the attack at the carbodiimide would be significantly slowed down compared to free uncoordinated aniline. However, this negative effect of palladium coordination to aniline should be overcompensated by coordination of carbodiimide to palladium in close proximity to aniline in fixed geometry as indicated in the presumed intermediates **6a**–**c**. There are precedents in the literature [22] showing that palladium(II) can interact weakly with accumulated carbodiimide bonds, but this type of complex is typically very labile and difficult to isolate under the reaction conditions due the presence of an aniline excess, and for this reason we have been unable to isolate labile intermediates **6a**–**c**. This preassociation between complexes **3a**–**c** and carbodiimide **2** to form intermediates **6a**–**c** would make easier the key step of C=N insertion leading to guanidines (Scheme 4).

Alternatively, it can be also envisioned that the acidity of hydrogen atoms bonded to nitrogen in intermediates **3a**–**c** increases sufficiently to protonate the nitrogen atom of the carbodiimide that subsequently would be activated to accept the nucleophilic attack of the resulting anilide anion or aniline (see Scheme 4). This mechanism would be similar to that accepted for peptide-bond formation mediated by carbodiimides [23–26]. It can also be possible that these two steps, i.e., protonation and nucleophilic attack occur in a quasi-concerted manner around the intermediates **6a**–**c**.

## Conclusion

In conclusion, we show the possibility to isolate and characterize palladium complexes by performing some reactions using large amounts of palladium salts. The structures of these complexes shed light onto the reaction mechanism of the palladium-catalyzed reaction. In this case, we have applied this methodology to isolate and characterize bis(anilino)- and bis(guanidino)palladium complexes that are proposed to be reaction intermediates, together with the still not isolated aniline–carbodiimide palladium complex **6**, in the mechanism of the guanylation of anilines. Our study opens the way to apply a similar methodology to study the reaction mechanism of other catalytic reactions.

## Supporting Information

File 1 Experimental details of preparation, isolation and full characterization of new palladium compounds **3a**–**c**, **4a**–**c** as well as guanidine compounds **5a**,**b**, including IR, NMR, ESIMS and GC–MS spectra for new compounds.

File 2 X-Ray structure analysis data for **4a** (CCDC-931786, **4b** (CCDC-931787), **4c** (CCDC-931788) and **5a** (CCDC-931789) are given. These data can also be obtained free of charge from The Cambridge Crystallographic Data Centre via http://www.ccdc.cam.ac.uk/data_request/cif.
