# Supplementary material for: Isolation and X-ray characterization of palladium–N complexes in the guanylation of aromatic amines. Mechanistic implications
Source: Beilstein J Org Chem. 2013 Jul 22;9:1455–62. doi: 10.3762/bjoc.9.165 (PMC3740567; doi:10.3762/bjoc.9.165)

Supporting Information

for

Isolation and X-ray characterization of palladium–N complexes in the guanylation of aromatic amines. Mechanistic implications

Abdessamad Grirrane*[1], Hermenegildo Garcia*[1], and Eleuterio Álvarez[2]

Address: [1]Instituto Universitario de Tecnología Química CSIC-UPV, Universidad Politécnica de Valencia, Av. De los Naranjos s/n, 46022 Valencia, Spain and [2]Instituto de Investigaciones Químicas CSIC-US, Departamento de Química Inorgánica, Av. Américo Vespucio 49, 41092 Sevilla, Spain

Email: Abdessamad Grirrane* - grirrane@itq.upv.es; Hermenegildo Garcia*- hgarcia@qim.upv.es

*Corresponding author

Experimental details of preparation, isolation and full characterization of new palladium compounds 3a–c, 4a–c as well as guanidine compounds 5a,b, including IR, NMR, ESIMS and GC–MS spectra for new compounds

Contents

Experimental Section S3

- Isolation of *trans*-dichlorobis(4-methoxyanilino-*κN*)palladium(II) complex (**3a**)
- Isolation of *trans*-dichlorobis(3,4-methylendioxyanilino-*κN*)palladium(II) (**3b**)
- Isolation of *trans*-dichlorobis(2-fluoro-4-iodoanilino-*κN*)palladium(II) (**3c**)
- Isolation of *trans*-dichlorobis[1,3-diisopropyl-2-(4-methoxyphenyl)guanidino-*κN*(aryl)]palladium(II) complex (**4a**)
- Isolation of *trans*-dichlorobis[1,3-diisopropyl-2-(3,4-methylendioxyphenyl)guanidino-*κN*(aryl)]palladium(II) complex (**4b**)
- Isolation of *trans*-dichlorobis[1,3-diisopropyl-2-(2-fluoro-4-iodophenyl)guanidino-*κN*(aryl)]palladium(II) complex (**4c**)
- Preparation of 1,3-diisopropyl-2-(4-methoxyphenyl)guanidine (**5a**)
- Preparation of 1,3-diisopropyl-2-(3,4-methylendioxyphenyl)guanidine (**5b**)
- Preparation of 2-(2-Fluoro-4-iodophenyl)-1,3-diisopropylguanidine (**5c**)

General Information about crystal X-ray structure analysis S9
IR spectrum of complex **3a** S10
IR spectrum of complex **3b** S11
IR spectrum of complex **3c**. S12
Solid state ^{13}C NMR spectrum of complex **3a** S13
Solid state ^{13}C NMR spectrum of complex **3b** S14
Solid state ^{13}C NMR spectrum of complex **3c** S15
Table S1: Crystal data and structure refining for **4a** S16
ORTEP drawing and crystal packing of **4a** S17
Spectral-Data ^{1}H, ^{13}C NMR for isolated compound (**4a**) S18
ESIMS *m*/*z* data for **4a** S20
Table S2: Crystal data and structure refining for **4b** S21
ORTEP drawing and crystal packing of **4b** S22
Spectral-Data ^{1}H, ^{13}C NMR for isolated compound (**4b**) S23
ESIMS *m*/*z* data for **4b** S25
Table S3: Crystal data and structure refining for **4c** S26
ORTEP drawing and crystal packing of **4c** S27
Spectral-Data ^{1}H, ^{19}F, ^{13}C NMR for isolated compound **4c** S28
ESIMS *m*/*z* data for **4c** S31
Table S4: Crystal data and structure refining for **5a** S32
ORTEP drawing and crystal packing of **5a** S33
Spectral-Data ^{1}H, ^{13}C NMR for isolated compound (**5a**) S34
ESIMS *m*/*z* data for **5a** S36
Spectral-Data ^{1}H, ^{13}C NMR for isolated compound (**5b**) S37
GC–MS *m*/*z* data for **5b** S39

Experimental Section:

General: All reactions were carried out under argon in dried solvent using a Solvent Purification System (SPS). ^{1}H NMR spectra were recorded on a 300 MHz spectrometer. Chemical shifts of ^{1}H signals are reported in ppm using the solvent peak as the internal standard ($CHDCl_2$: 5.27 ppm). Data are reported as follows: chemical shift, integral, multiplicity (s = singlet, d = doublet, dd = double doublets, br = broad, m = multiplet), coupling constants (Hz) and assignment. Chemical shifts of ^{13}C are reported also in ppm using the solvent peak as the internal standard ($CHDCl_2$: 53.84 ppm). Solid-state ^{13}C NMR spectra were recorded on a 400 MHz spectrometer.

Gas chromatography (GC) was performed in a Varian 3900 apparatus equipped with a TRB-5MS column (5% phenyl, 95% polymethylsiloxane, 30 m, 0.25 mm × 0.25 µm, Teknokroma). GC–MS analyses were performed on an Agilent spectrometer equipped with the same column as the GC and operated under the same conditions. ESIMS were performed on an Agilent Esquire 6000 instrument. Elemental analyses were performed on a EuroEA Elemental Analyser Eurovector. IR spectra were measured in a Nicolet 710 FT IR spectrometer.

Isolation of *trans*-dichlorobis(4-methoxyanilino-*κN*)palladium(II) complex (3a):

Dichloromethane (1.5 mL) was added to a mixture of $PdCl_2(NCMe)_2$ complex (0.039 g, 0.15 mmol) and 4-methoxyaniline (**1a**) (0.037 g, 0.30 mmol). The solution was stirred at 60 °C under argon atmosphere (pressure 2 bar). Rapid formation of a pale yellow precipitate corresponding to **3a** was observed. The stirring was continued for 40 h and then the pale yellow solid was collected by filtration and washed with cold dichloromethane (3 × 2 mL) and dried at 60 °C under reduced pressure (0.058 g, 91% yield).

Elemental analysis, calcd for: $C_{14}H_{18}Cl_2N_2O_2Pd$ (%): C, 39.69; H, 4.28; N, 6.61. Found: C, 39.55; H, 4.56; N, 6.62.

IR (KBr): ν (cm^{-1}) = 3451 (br), 3284 (s), 3201 (s), 3117 (m), 3015 (w), 2834 (w), 1575 (m), 1510 (vs), 1464 (m), 1249 (vs), 1175 (m), 1123 (m), 1027 (m), 827 (m), 739 (w), 559 (w) (see Figure S1 for details).

^{13}C NMR (105 MHz, solid sate): δ (ppm) = 156.37, 134.84, 125.79, 123.71, 117.20, 110.70, 54.21 (see Figure S4 for details).

Isolation of *trans*-dichlorobis(3,4-methylendioxyanilino-*κN*)palladium(II) (3b):

Dichloromethane (1.5 mL) was added to a mixture of $PdCl_2(NCMe)_2$ complex (0.039 g, 0.15 mmol) and 3,4-methylendioxyaniline (**1b**) (0.041 g, 0.30 mmol). The solution was stirred at 60 °C under argon atmosphere (pressure 2 bar). Rapid formation of a green grey precipitate corresponding to **3b** was observed. The stirring was continued for 40 h and then the green grey solid was collected by filtration and washed with cold dichloromethane (3 × 2 mL) and dried at 60 °C under reduced pressure (0.060 g, 88% yield).

Elemental analysis calcd for: $C_{14}H_{14}Cl_2N_2O_4Pd$ (%): C, 37.23; H, 3.12; N, 6.20. Found: C, 37.09; H, 3.05; N, 6.25.

IR (KBr): ν (cm^{-1}) = 3461 (br), 3279 (s), 3214 (s), 3115 (w), 2902 (w), 1570 (m), 1504 (s), 1491 (vs), 1460 (m), 1259 (s), 1191 (w), 1118 (m), 1039 (m), 941 (w), 922 (m), 856 (w), 813 (m), 623 (w) (see Figure S2 for details).

^{13}C NMR (105 MHz, solid sate): δ (ppm) = 147.78, 144.45, 136.17, 117.71, 109.17, 102.71 (see Figure S5 for details).

Isolation of *trans*-dichlorobis(2-fluoro-4-iodoanilino-*κN*)palladium(II) (3c):

Dichloromethane (1.5 mL) was added to a mixture of $PdCl_2(NCMe)_2$ complex (0.039 g, 0.15 mmol) and 2-fluoro-4-iodoaniline (**1c**) (0.071 g, 0.30 mmol). The solution was stirred at 60 °C under argon atmosphere (pressure 2 bar). Rapid formation of a yellow precipitate corresponding to **3c** was observed. The stirring was continued for 40 h and then the yellow solid was collected by filtration and washed with cold dichloromethane (3 × 2 mL) and dried at 60 °C under reduced pressure (0.091 g, 93% yield).

Elemental analysis calcd for: $C_{12}H_{10}Cl_2F_2I_2N_2Pd$ (%): C, 22.13; H, 1.55; N, 4.30. Found: C, 22.20; H, 1.70; N, 4.33.

IR (KBr): ν (cm^{-1}) = 3465 (br), 3254 (s), 3166 (m), 3147 (m), 3193 (s), 1591 (m), 1567 (s), 1483 (vs), 1406 (m), 1272 (w), 1140 (m), 1100 (m), 873 (m), 860 (m), 816 (m), 776 (w), 582 (w), 439 (w) (see Figure S3 for details).

^{13}C NMR (105 MHz, solid sate): δ (ppm) = 154.19, 151.59, 134.62, 127.65, 126.04, 90.44 (see Figure S6 for details).

Isolation of *trans*-dichlorobis[1,3-diisopropyl-2-(4-methoxyphenyl)guanidino-*κN*(aryl)]palladium(II) complex (4a):

A mixture of complex $PdCl_2(NCMe)_2$ (0.039 g, 0.15 mmol), 4-methoxyaniline (**1a**) (0.037 g, 0.30 mmol) and *N*,*N*'-diisopropylcarbodiimide (**2**) (0.040 g, 0.32 mmol) was dissolved in dichloromethane (1.5 mL). The solution was stirred at 60 °C under argon atmosphere (pressure 2 bar), whereby the formation of a pale yellow precipitate corresponding to the previous $PdCl_2$bis(4-methoxyanilino) (**3a**) complex took place. The stirring was continued for 50 h and during this time the majority of the initial complex **3a** formed was reacted with the carbodiimide **2**. Then, the resulting mixture was filtered and the supernatant was evaporated to dryness under vacuum. The crude product was washed with cold diethyl ether (3 × 1 mL), and an orange-red solid material corresponding to complex **4a** was obtained and dried at 60 °C under vacuum (0.088 g, 86% yield). Crystals of **4a** suitable for X-ray crystallography (see Table S1 and Figure S7 for details) were obtained by recrystallization of the orange-red precipitate in a mixture of dichloromethane/toluene (1/1).

Elemental analysis calculated for $C_{28}H_{46}Cl_2N_6O_2Pd$ (**4a**) (%): C, 49.75; H, 6.86; N, 12.43. Found: C, 49.47; H, 7.50; N, 12.25.

^{1}H NMR (300 MHz, CD_2Cl_2): δ (ppm) = 7.26 (4 H; *m*-C_6H_4); 6.79 (4 H; *o*-C_6H_4), 4.59 (br, 4 H; NH), 3.74 (s, 6 H; CH_3), 3.58 (m, 4 H; CH), 1.07 (d, 24 H; CH_3) (see Figure S8 for details).

^{13}C NMR (75 MHz, CD_2Cl_2): δ (ppm) = 157.54, 157.42, 139.65, 128.41, 114.60, 55.77, 47.26, 23.81 (see Figure S9 for details).

ESIMS (+MS) *m*/*z*: 639.3 amu for $[C_{28}H_{46}Cl_2N_6O_2Pd$ (**4a**) – $Cl^-]^+$. Peak exhibited the isotopic distribution characteristic of the presence of Pd and Cl (see Figure S10 for details).

ESIMS (–MS) *m*/*z*: 711.2 amu for $[C_{28}H_{46}Cl_2N_6O_2Pd$ (**4a**) + $Cl^-]^-$. Peak exhibited the isotopic distribution characteristic of the presence of Pd and Cl (see Figure S10 for details).

Isolation of *trans*-dichlorobis[1,3-diisopropyl-2-(3,4-methylendioxyphenyl)guanidino-*κN*(aryl)]palladium(II) complex (4b):

A mixture of complex $PdCl_2(NCMe)_2$ (0.039 g, 0.15 mmol), 3,4-methylendioxyaniline (**1b**) (0.041 g, 0.30 mmol) and *N*,*N*'-diisopropylcarbodiimide (**2**) (0.040 g, 0.32 mmol)

was dissolved in dichloromethane (1.5 mL). The solution was stirred at 60 °C under an argon atmosphere (pressure 2 bar), whereby a green grey precipitate corresponding to the previous $PdCl_2$bis(3,4-methylendioxyanilino) (**3b**) complex was formed. The stirring was continued for 60 h and during this time the majority of the initial complex **3b** formed was reacted with the carbodiimide **2**. Then, the resulting mixture was filtered and the supernatant was evaporated to dryness under vacuum. The crude product was washed with cold diethyl ether (3 × 1 mL), and an orange-red solid material corresponding to complex **4b** was obtained and dried at 60 °C under vacuum (0.082 g, 78% yield). Crystals of **4b** suitable for X-ray crystallography (see Table S2 and Figure S11 for details) were obtained by recrystallization of the orange-red precipitate in a mixture of dichloromethane/diethyl ether (1/1).

Elemental analysis calculated for $C_{28}H_{42}Cl_2N_6O_4Pd$ (**4b**)·1/2($C_4H_{10}O$) (%): C, 48.62; H, 6.39; N, 11.34. Found: C, 48.81; H, 6.71; N, 11.90.

^{1}H NMR (300 MHz, CD_2Cl_2): δ (ppm) = 6.91 (d, 2 H; *m*-C_6H_3), 6.83-6.80 (dd, 2 H; *o*-C_6H_3), 6.69 (d, 2 H; *o*-C_6H_3), 5.91 (s, 4 H; CH_2), 4.65 (br, 4 H; NH), 3.60 (m, 4 H; CH), 1.10 (d, 24 H; CH_3) (see Figure S12).

^{13}C NMR (75 MHz, CD_2Cl_2): δ (ppm) = 157.56, 148.26, 145.34, 140.99, 120.38, 108.66, 108.51, 101.84, 47.24, 23.82 (see Figure S13).

ESIMS (+MS) *m*/*z*: 667.2 amu for [$C_{28}H_{42}Cl_2N_6O_4Pd$ (**4b**) – Cl^-]$^+$. Peak exhibited the isotopic distribution characteristic of the presence of Pd and Cl (see Figure S14 for details).

ESIMS (–MS) *m*/*z*: 739.1 amu for [$C_{28}H_{42}Cl_2N_6O_4Pd$ (**4b**) + Cl^-]$^-$. Peak exhibited the isotopic distribution characteristic of the presence of Pd and Cl (see Figure S14 for details).

Isolation of *trans*-dichlorobis[1,3-diisopropyl-2-(2-fluoro-4-iodophenyl)guanidino-*κN*(aryl)]palladium(II) complex (4c):

A mixture of complex $PdCl_2(NCMe)_2$ (0.039 g, 0.15 mmol), 2-fluoro-4-iodoaniline (**1c**) (0.071 g, 0.30 mmol) and *N*,*N*'-diisopropylcarbodiimide (**2**) (0.040 g, 0.32 mmol) was dissolved in dichloromethane (1.5 mL). The solution was stirred at 60 °C under an argon atmosphere (pressure 2 bar), whereby a yellow precipitate corresponding to the previous $PdCl_2$bis(2-fluoro-4-iodoanilino) (**3c**) complex was formed. The stirring was continued for 60 h and during this time the majority of the initial complex **3c** formed

was reacted with the carbodiimide **2**. Then, the resulting mixture was filtered and the supernatant was evaporated to dryness under vacuum. The crude product was washed with cold diethyl ether (3 × 1 mL), and a red solid material corresponding to complex **4c** was obtained and dried at 60 °C under vacuum (0.12 g, 88% yield).

Crystals of **4c** suitable for X-ray crystallography (see Table S3 and Figure S15 for details) were obtained by recrystallization of the red precipitate in a mixture of dichloromethane/toluene (1/1).

Elemental analysis calculated for $C_{26}H_{38}Cl_2F_2I_2N_6Pd$ (**4c**) (%): C, 34.55; H, 4.24; N, 9.30. Found: C, 35.17; H, 4.42; N, 9.28.

^1H NMR (300 MHz, CD_2Cl_2): δ (ppm) = 7.90 (t, 2 H; *m*-C_6H_3F), 7.47-7.45 (ddd, 2 H; *o*-C_6H_3F), 7.47-7.45 (dd, 2 H; *m*-C_6H_3F), 4.88 (br, 4 H; NH), 3.75-3.59 (m, 4 H; CH), 1.12 (d, 24 H; CH_3) (see Figure S16 for details)

^{19}F NMR (300 MHz, CD_2Cl_2): δ (ppm) = –118.62 (t, 2 F) (see Figure S17 for details).

^{13}C NMR (75 MHz, CD_2Cl_2): δ (ppm) = 158.40, 157.59, 155.04, 135.97, 135.81, 134.21, 134.16, 132.14, 132.11, 125.71, 125.41, 87.29, 87.19, 46,63, 23,72 (see Figure S18 for details).

ESIMS (+MS) *m*/*z*: 867.1 amu for [$C_{26}H_{38}Cl_2F_2I_2N_6Pd$ (**4c**) – Cl^-]$^+$. Peak exhibited the isotopic distribution characteristic of the presence of Pd and Cl (see Figure S19 for details).

ESIMS (–MS) *m*/*z*: 938.9 amu for [$C_{26}H_{38}Cl_2F_2I_2N_6Pd$ (**4c**), + Cl^-]$^-$. Peak exhibited the isotopic distribution characteristic of the presence of Pd and Cl (see Figure S19 for details).

Preparation of 1,3-diisopropyl-2-(4-methoxyphenyl)guanidine (**5a**):

A mixture of 4-methoxyaniline (**1a**) (0.25 mmol), *N*,*N*'-diisopropylcarbodiimide (**2**) (0.27 mmol) and complex $PdCl_2(NCMe)_2$ (0.01 mmol) as catalyst (Pd/substrate ratio 4 mol %) was suspended in toluene (1 mL), and then the flask was evacuated under vacuum and refilled with argon. The evacuation/refilling cycle was repeated three times (pressure 2 bar). The mixture was stirred at 130 °C for 15 h to obtain the maximum yield of **5a**, the reaction mixture was filtered and the solvent was removed under reduced pressure. The residue was sublimed under vacuum at 95 °C to provide, in a yield higher than 75%, colourless crystals of **5a** suitable for X-ray crystallography (see Table S4 and Figure S20 for details).

Elemental analysis calculated for $C_{14}H_{23}N_3O$ (%): C, 67.43; H, 9.30; N, 16.85. Found: C, 67.15; H, 9.75; N, 16.74.
^{1}H NMR (300 MHz, CD_2Cl_2): δ (ppm) = 6.75 (d, 2 H; *m*-C_6H_4), 6.65 (d, 2 H; *o*-C_6H_4), 3.72-3.68 (m, 2 H; CH), 3.70 (s, 3 H; CH_3), 3.54 (br, 2 H; NH), 1.08 (d, 12 H; CH_3) (see Figure S21 for details).
^{13}C NMR (75 MHz, CD_2Cl_2): δ (ppm) = 154.80, 150.72, 144.02, 124.22, 114.98, 55.74, 43.59, 23,51 (see Figure S22 for details).
ESIMS (+MS) *m*/*z*: 250.2 amu for [$C_{14}H_{23}N_3O$ (**5a**), H]$^+$ (see Figure S23 for details).

Preparation of 1,3-diisopropyl-2-(3,4-methylendioxyphenyl)guanidine (**5b**):
A mixture of 3,4-methylendioxyaniline (**1b**) (0.25 mmol), *N*,*N*'-diisopropylcarbodiimide (**2**) (0.27 mmol) and complex $PdCl_2(NCMe)_2$ (0.01 mmol) as catalyst (Pd/substrate ratio 4 mol %) was suspended in toluene (1 mL), then the flask was evacuated under vacuum and refilled with argon. The evacuation/refilling cycle was repeated three times (pressure 2 bar). The mixture was stirred at 130 °C for 17 h to obtain the maximum yield of **5b**, the reaction mixture was filtered and the solvent was removed under reduced pressure. The residue was sublimed under vacuum at 95 °C to provide, in yield higher than 70%, colourless crystals of **5b**.
Elemental analysis calculated for $C_{14}H_{21}N_3O_2$ (%): C, 63.85; H, 8.04; N, 15.96. Found: C, 63.57; H, 8.82; N, 16.16.
^{1}H NMR (300 MHz, CD_2Cl_2): δ (ppm) = 6.64 (d, 1 H; *m*-C_6H_3), 6.28 (d, 1 H; *o*-C_6H_3), 6.18-6.15 (dd, 1 H; *o*-C_6H_3), 5.83 (s, 2 H; CH_2), 3.8-3.6 (br and m, 4 H; NH and CH), 1.08 (d, 12 H; CH_3) (see Figure S24 for details).
^{13}C NMR (75 MHz, CD_2Cl_2): δ (ppm) = 150.96, 148.63, 145.23, 142.54, 115.36, 108.81, 105.06, 101.24, 43.68, 23.47 (see Figure S25 for details).
GC–MS *m*/*z*: 263.2 amu for [$C_{14}H_{21}N_3O_2$ (**5b**)] (see Figure S26 for details).

Preparation of 2-(2-fluoro-4-iodophenyl)-1,3-diisopropylguanidine (**5c**) [1]:
A mixture of 2-fluoro-4-iodoaniline (**1c**) (0.25 mmol), *N*,*N*'-diisopropylcarbodiimide (**2**) (0.27 mmol) and complex $PdCl_2(NCMe)_2$ (0.01 mmol) as catalyst (Pd/substrate ratio 4 mol %) was suspended in toluene (1 mL). Then the flask was evacuated under vacuum and refilled with argon. The evacuation/refilling cycle was repeated three times

(pressure 2 bar). The mixture was stirred at 130 °C for 16 h to obtain the maximum yield of **5c**, the reaction mixture was filtered and the solvent was removed under reduced pressure. The residue was sublimed under vacuum at 95 °C to provide in yield higher than 72% colourless crystals of **5c**.

X-ray crystal structure analysis for 4a, 4b, 4c and 5a.

Crystals of suitable size for X-ray diffraction analysis were coated with dry perfluoropolyether and mounted on glass fibers and fixed in a cold nitrogen stream (T = 100 K) to the goniometer head. Data collections were performed on a Bruker-Nonius X8Apex-II CCD diffractometer, using monochromatic radiation $\lambda(MoK_{\alpha})$ = 0.71073 Å, by means of ω and φ scans with a width of 0.50 degree. The data were reduced (SAINT) [2] and corrected for absorption effects by the multi-scan method (SADABS) [3]. The structures were solved by direct methods (SIR-2002) [4] and refined against all F^2 data by full-matrix least-squares techniques (SHELXTL-6.12) [5] minimizing $w[F_o^2-F_c^2]^2$. All the nonhydrogen atoms were refined anisotropically, while C–H hydrogen atoms were placed in geometrically calculated positions using a riding model. The N–H hydrogen atoms were localized by difference Fourier maps and refined fixing their bond lengths at the end of the refinement.

References

[1] For full characterization details of compound **5c**, see our recent published work: *Chem. Eur. J.* **2012**, 18, 14934-14938.

[2] Bruker (2007). *APEX2*. Bruker AXS Inc., Madison, Wisconsin, USA.

[3] Bruker (2001). *APEX2*. Bruker AXS Inc., Madison, Wisconsin, USA.

[4] C. M. Burla, M. Camalli, B. Carrozzini, G. L. Cascarano, C. Giacovazzo, G. Poliori, R. Spagna, *SIR2002*: the program; (2003). *J. Appl. Cryst.* **36**, 1103–1103.

[5] G. M. Sheldrick, *Acta Cryst.*, 2008, A**64**, 112.

Figure S1: IR spectrum of complex (**3a**).

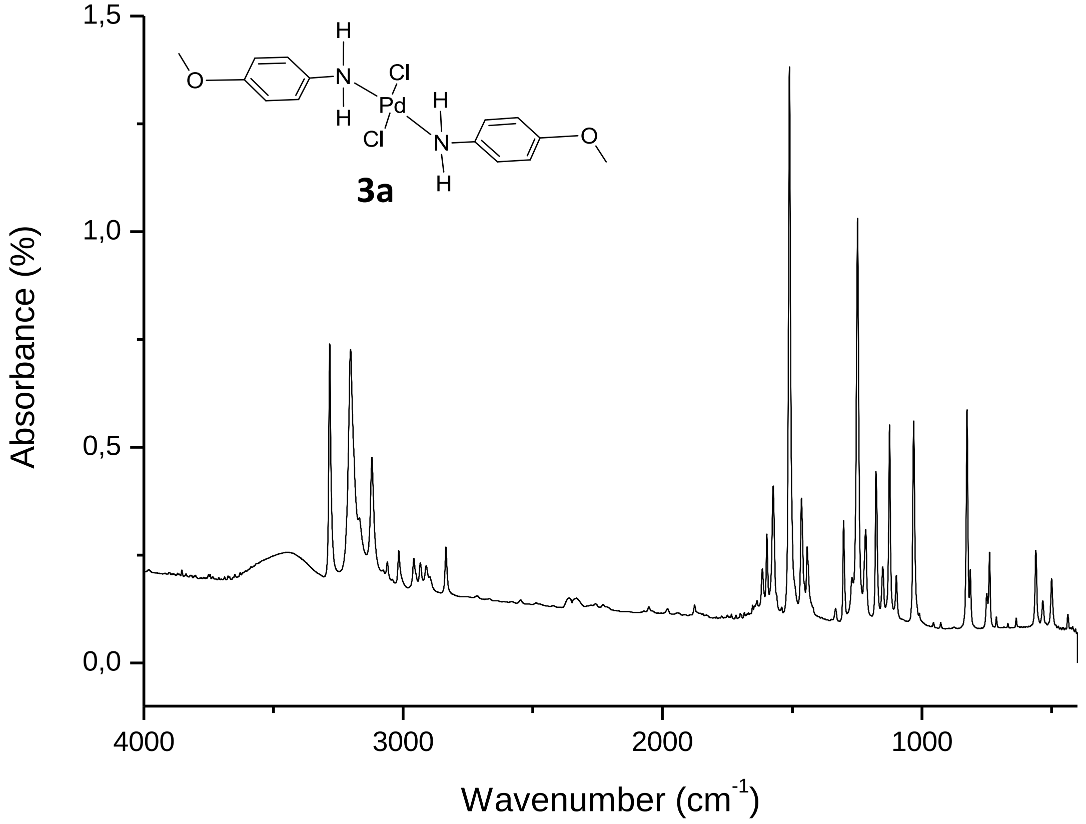

Figure S2: IR spectrum of complex (**3b**).

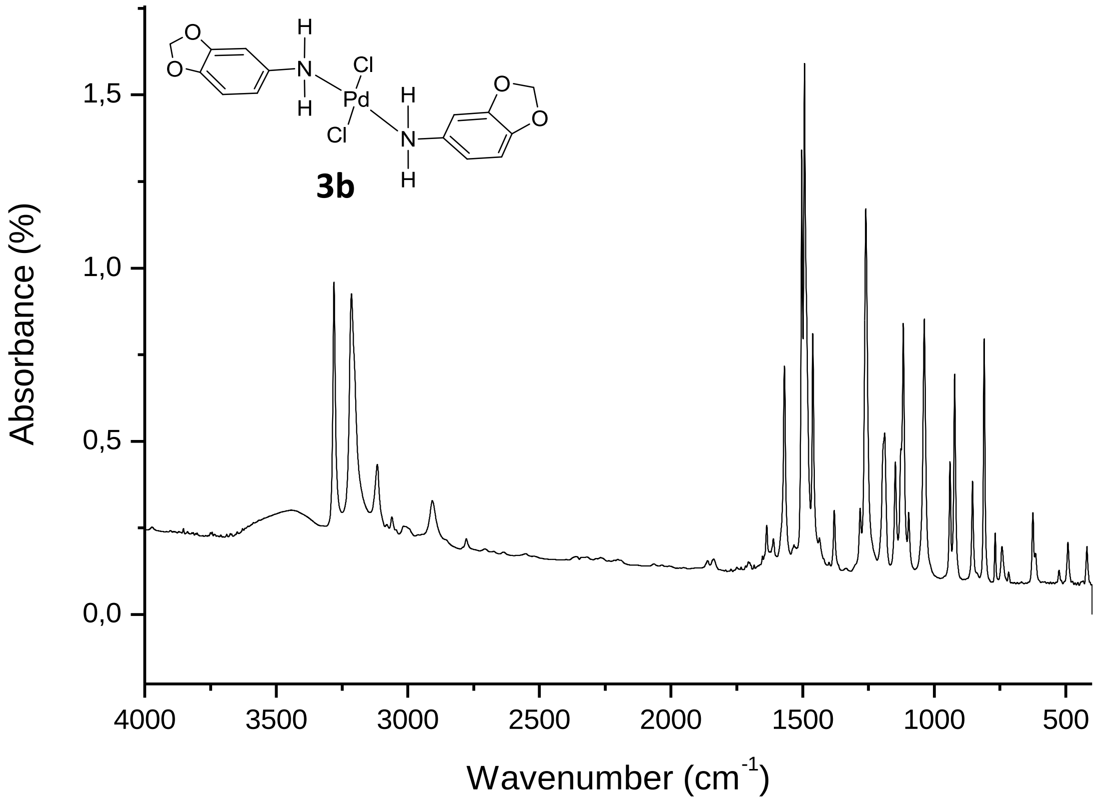

Figure S3: IR spectrum of complex (**3c**).

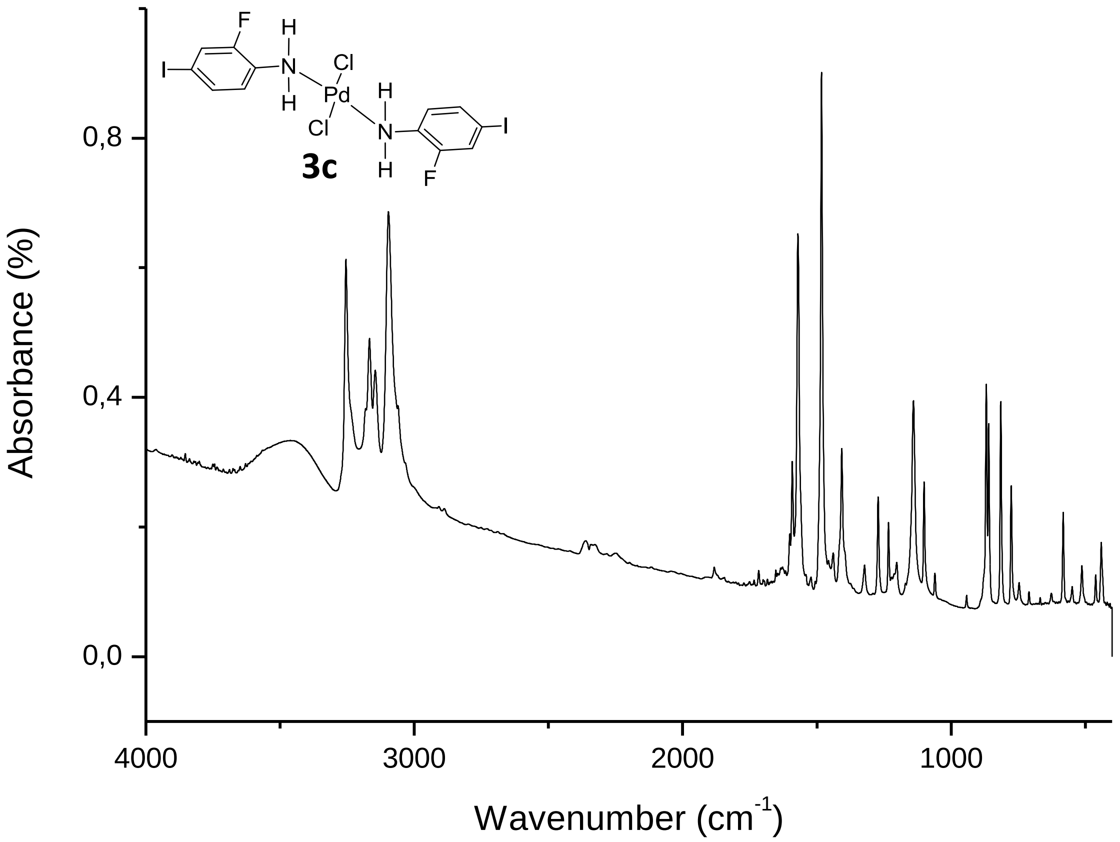

Figure S4: Solid state ^{13}C NMR spectrum of complex **3a**.

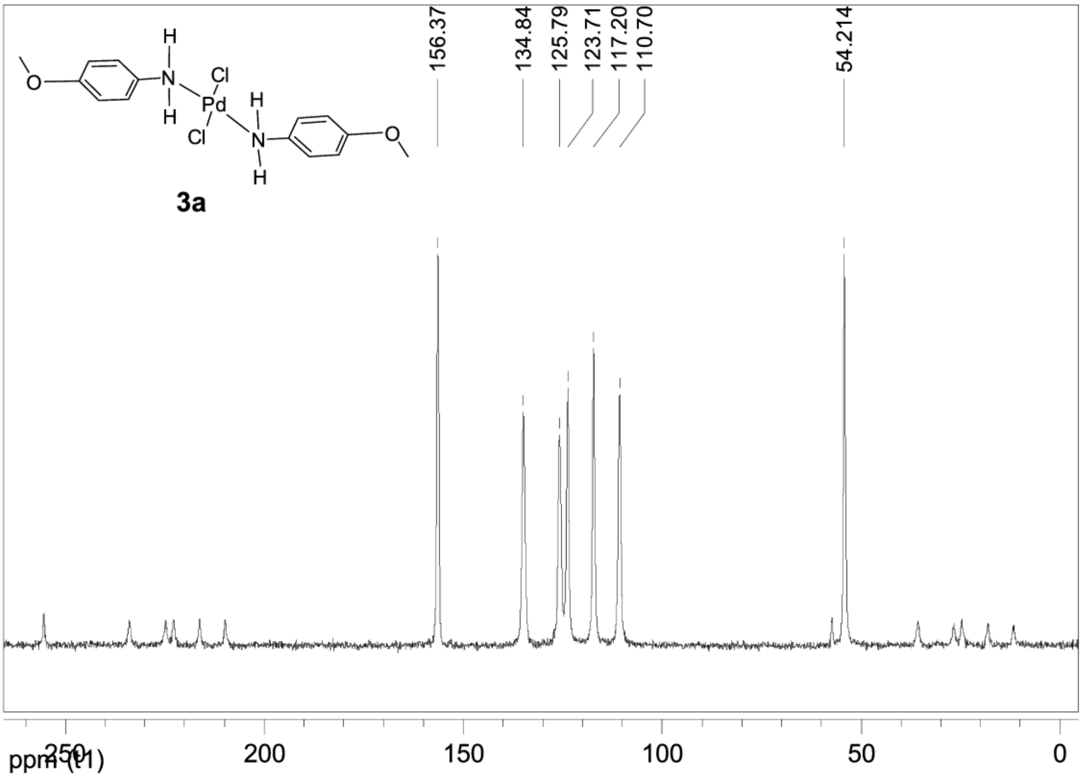

Magnification of the ^{13}C NMR peaks (**3a**):

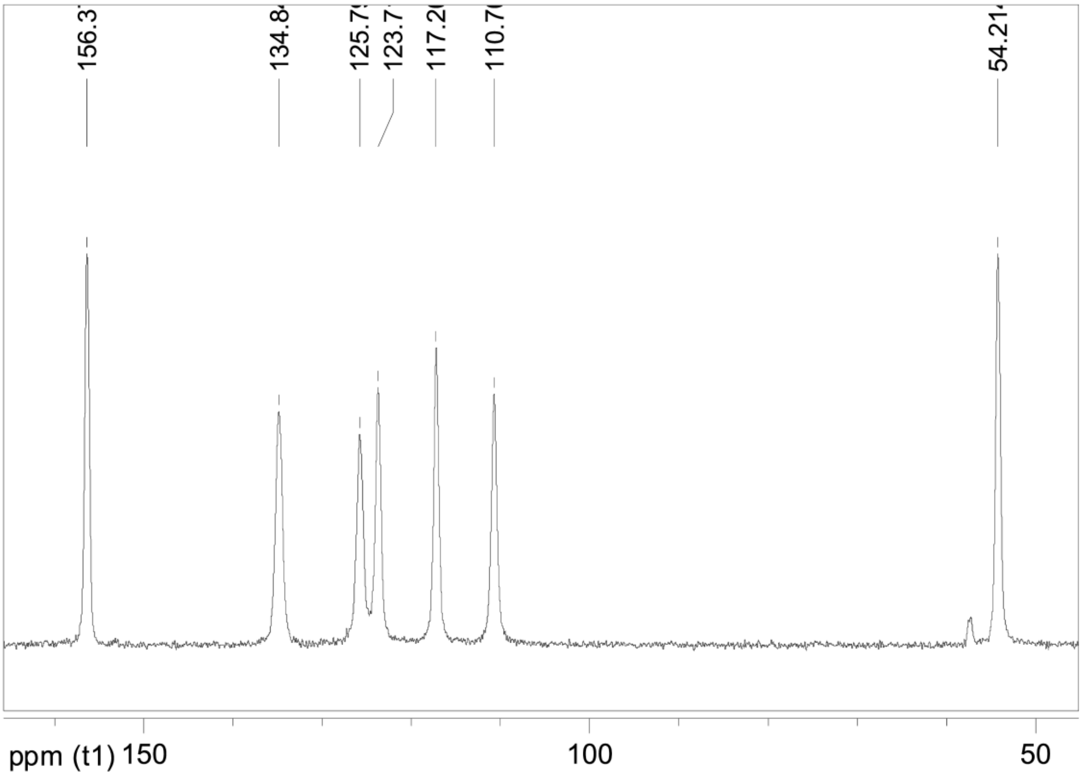

Figure S5: Solid state ^{13}C NMR spectrum of complex **3b**.

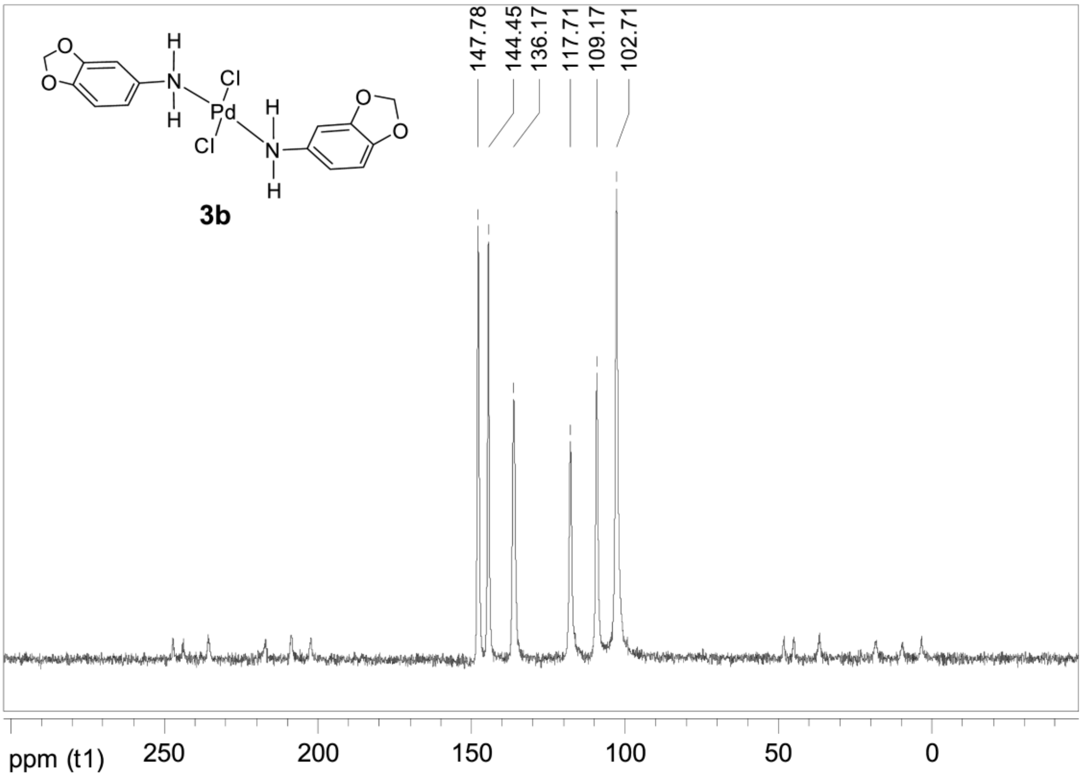

Magnification of the ^{13}C NMR peaks (**3b**):

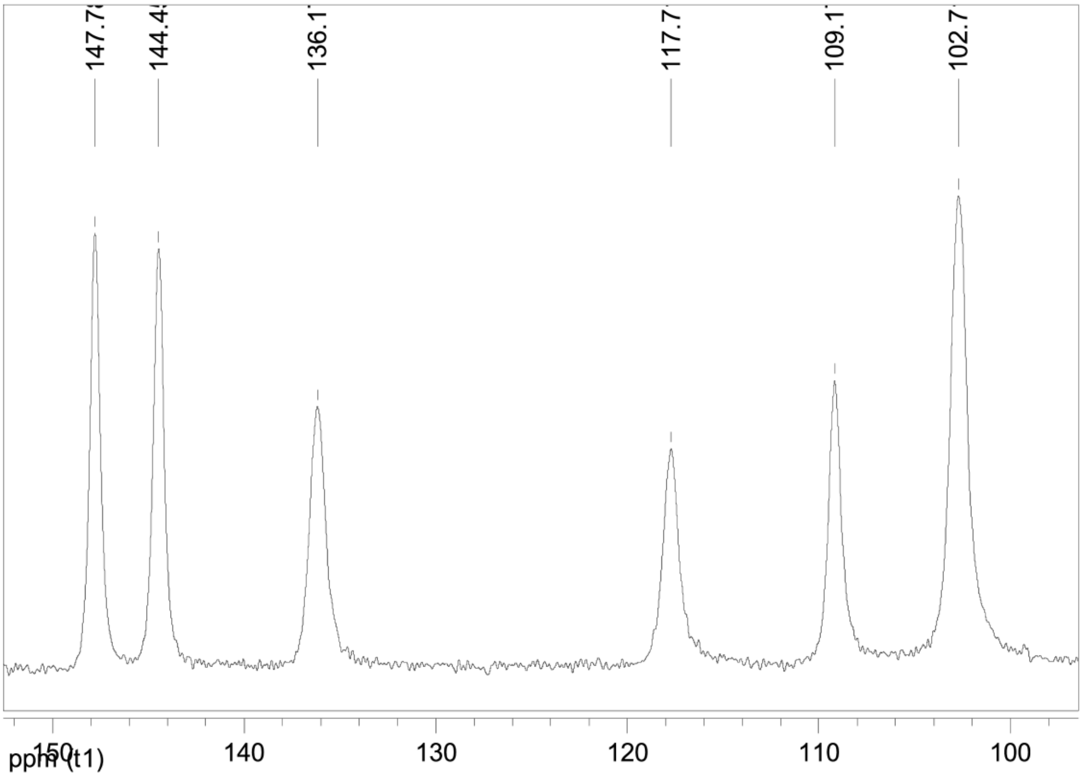

Figure S6: Solid state ^{13}C NMR spectrum of complex **3c**.

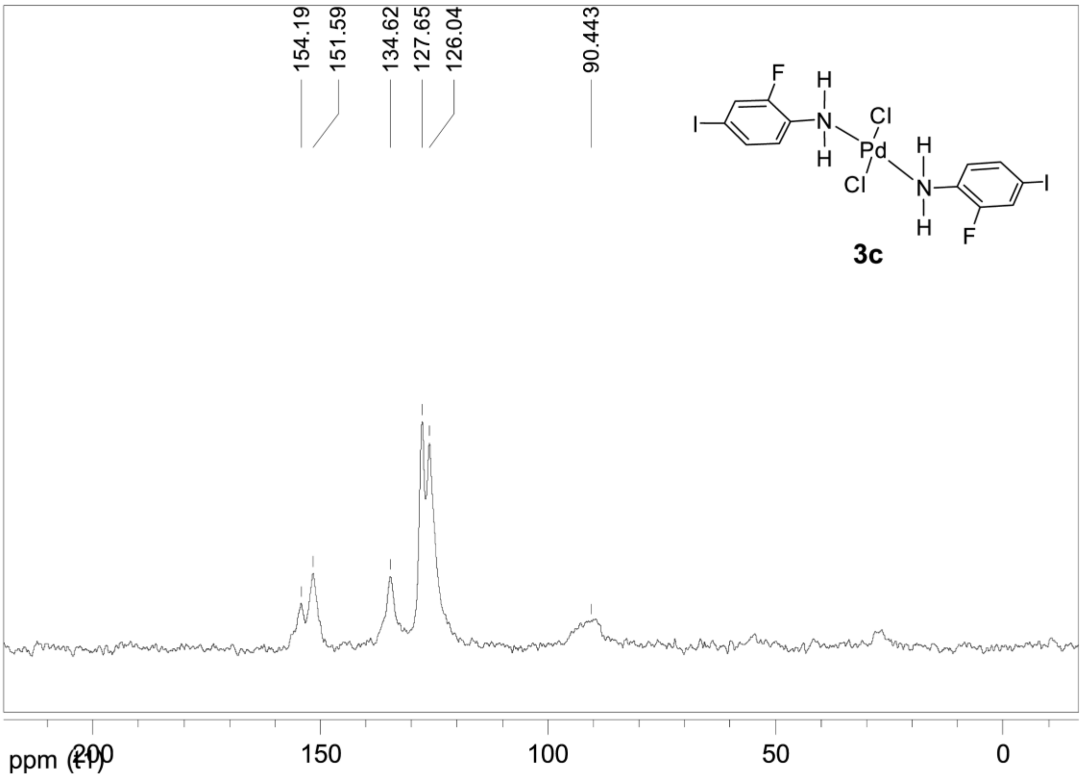

Magnification of the ^{13}C NMR peaks (**3c**):

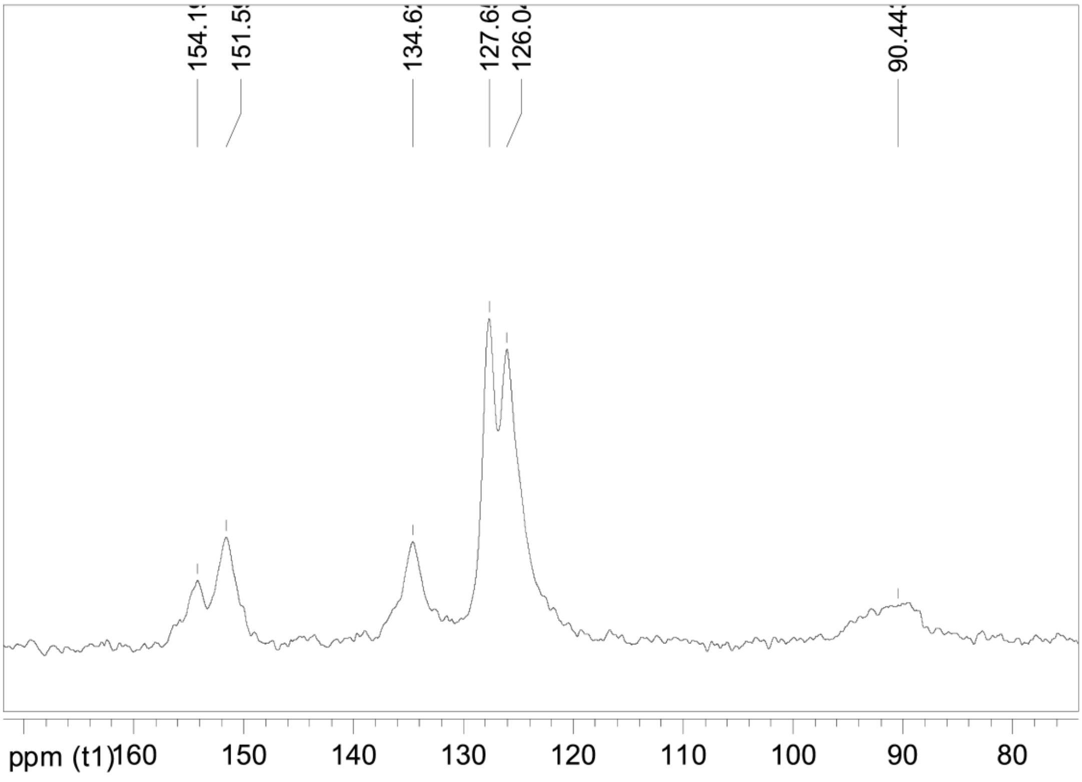

Table S1: Crystal data and structure refining for **4a:**

Empirical formula	$C_{28}H_{46}Cl_2N_6O_2Pd$	
Formula weight	676.01	
Temperature	173(2) K	
Wavelength	0.71073 Å	
Crystal system	Monoclinic	
Space group	P 2_1/c	
Unit cell dimensions	a = 11.3420(5) Å	α= 90°.
	b = 18.0868(7) Å	β= 95.4700(10)°.
	c = 7.6720(3) Å	γ = 90°.
Volume	1566.67(11) $Å^3$	
Z	2	
Density (calculated)	1.433 Mg/m^3	
Absorption coefficient	0.798 mm^{-1}	
F(000)	704	
Crystal size	0.40 x 0.20 x 0.15 mm^3	
Theta range for data collection	1.80 to 30.52°.	
Index ranges	-16<=h<=16, -15<=k<=25, -10<=l<=10	
Reflections collected	20959	
Independent reflections	4738 [R(int) = 0.0188]	
Completeness to theta = 30.52°	99.2%	
Absorption correction	Semi-empirical from equivalents	
Max. and min. transmission	0.8869 and 0.8258	
Refinement method	Full-matrix least-squares on F^2	
Data / restraints / parameters	4738 / 0 / 189	
Goodness-of-fit on F^2	1.060	
= 0.0641		
Largest diff. peak and hole	1.051 and -0.611 e	
Final R indices [I>2sigma(I)]	R1 = 0.0239, wR2 = 0.0627	
R indices (all data)	R1 = 0.0271, wR2.$Å^{-3}$	

Figure S7: ORTEP drawing of **4a**.

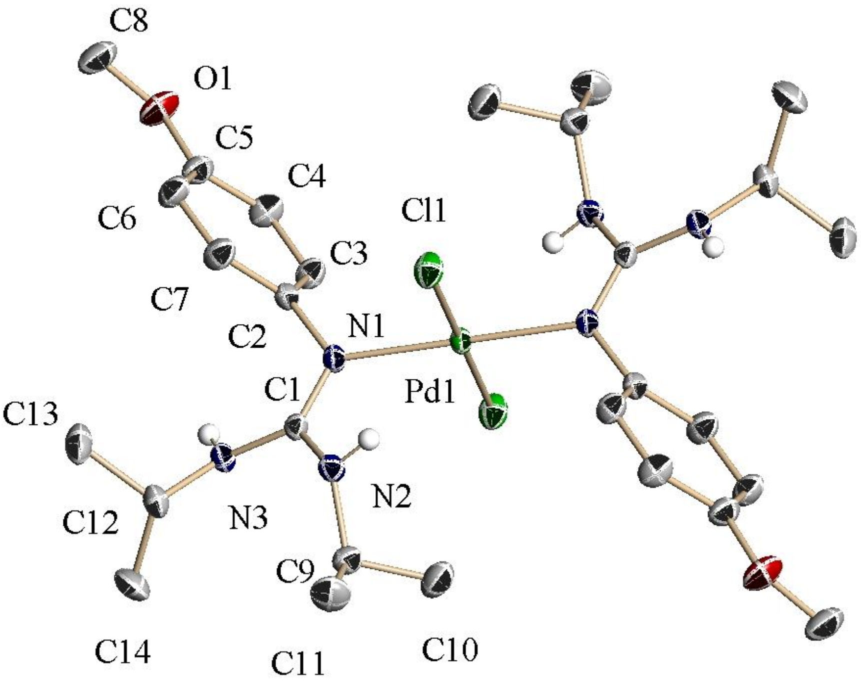

Crystal packing detail of **4a** viewed along the a-axis showing the presence of inter- and intramolecular hydrogen bonds between Cl and H (NH groups) atoms:

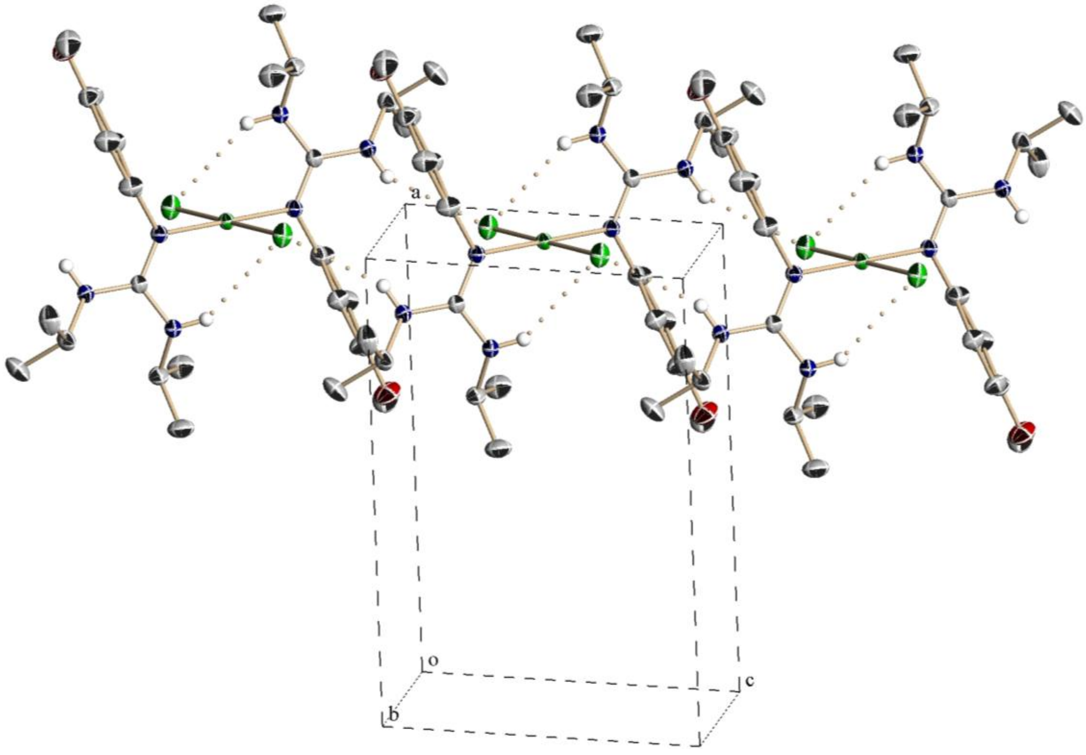

Figure S8: ^{1}H NMR spectrum in CD_2Cl_2 for isolated complex **4a**.

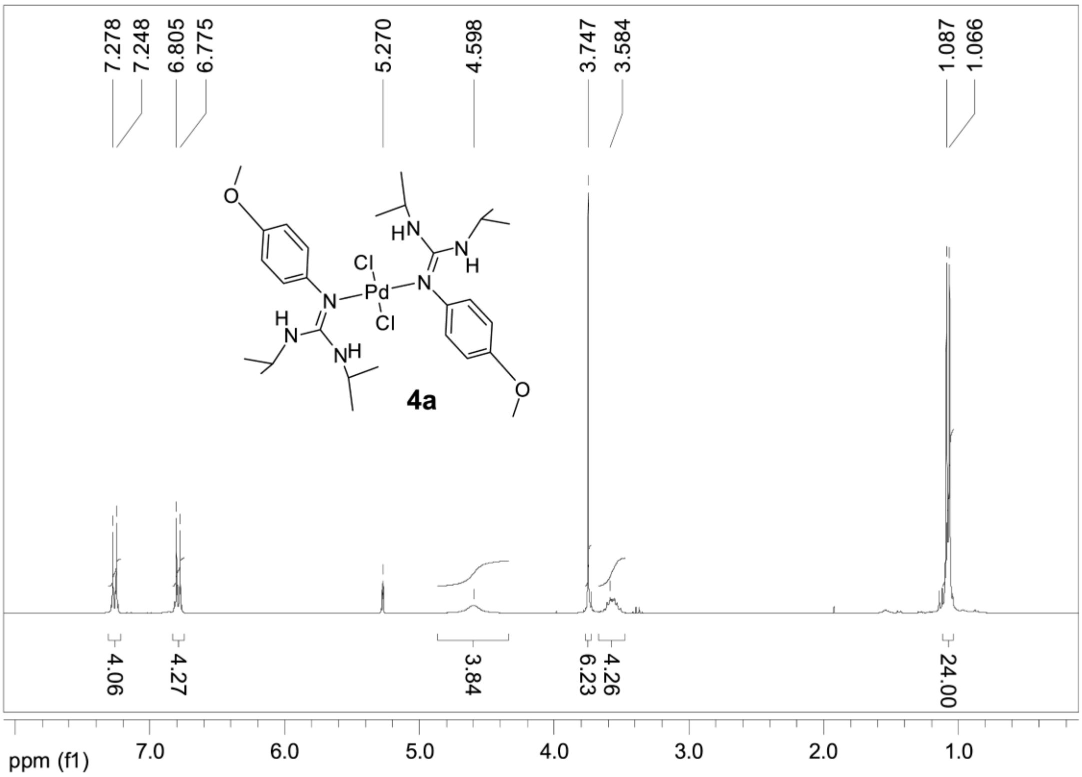

Magnification of the ^{1}H NMR peaks (**4a**):

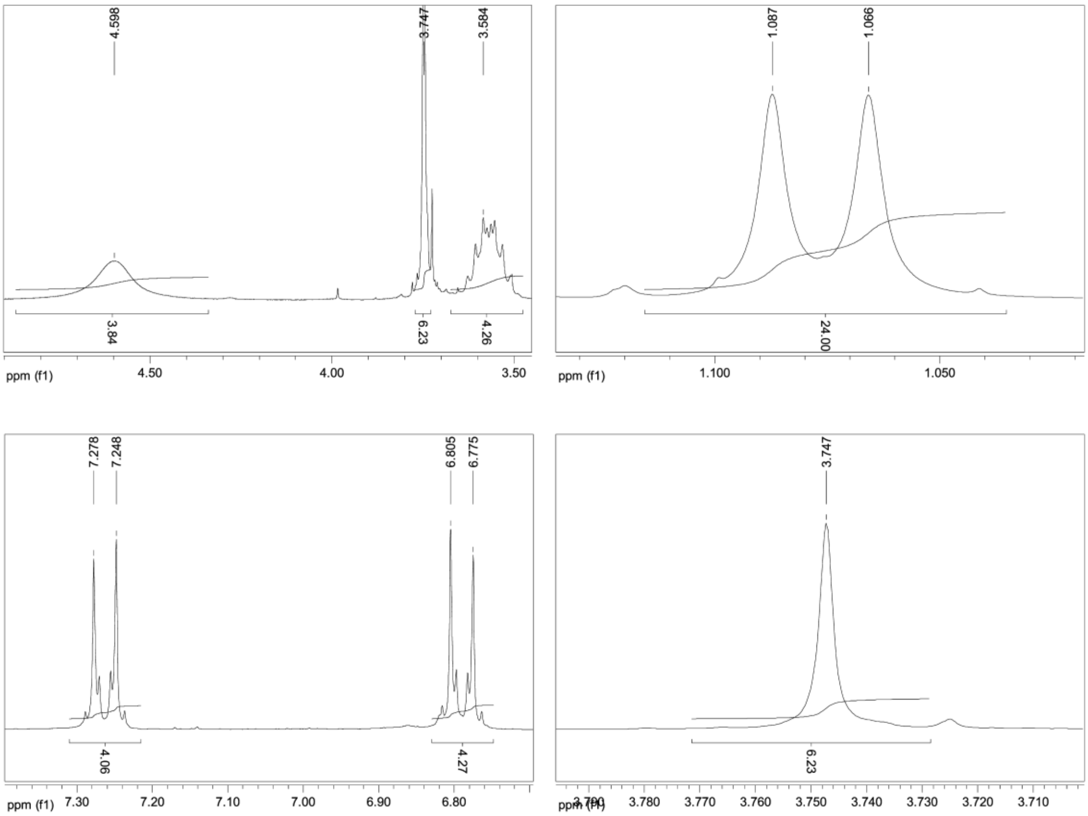

Figure S9: ^{13}C NMR spectrum in CD_2Cl_2 for isolated complex (**4a**).

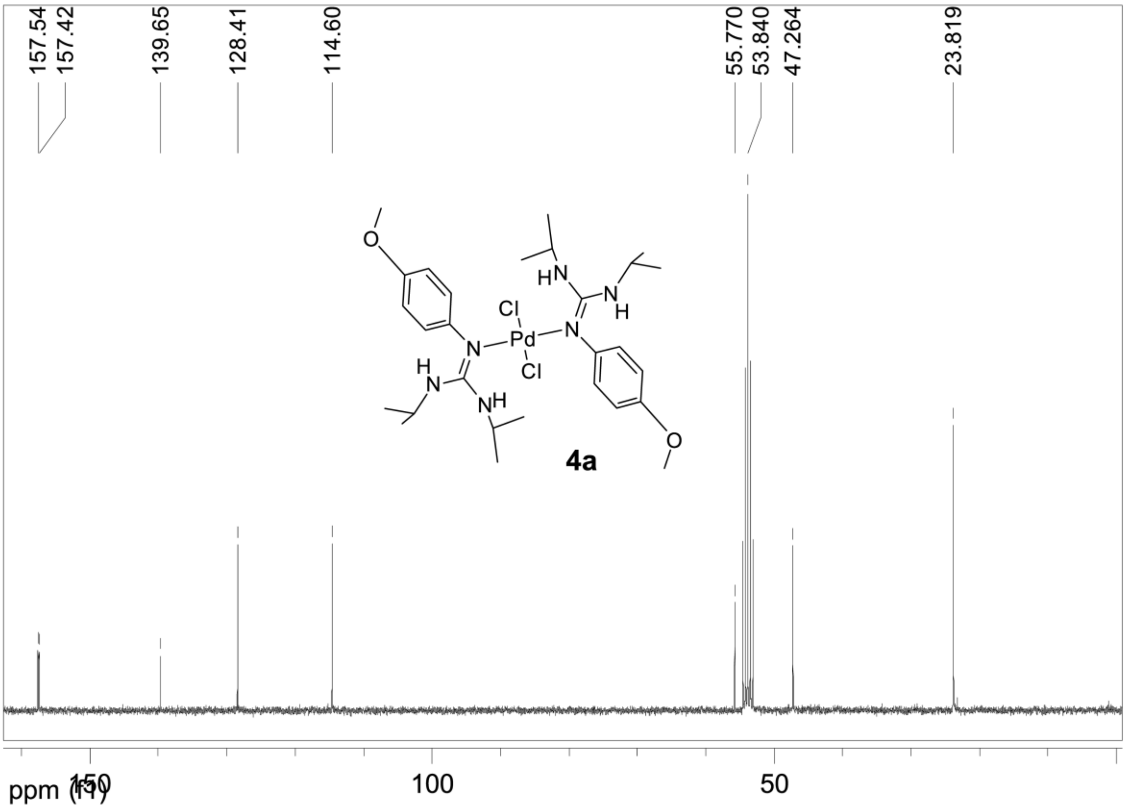

Magnification of the ^{13}C NMR peaks (**4a**).

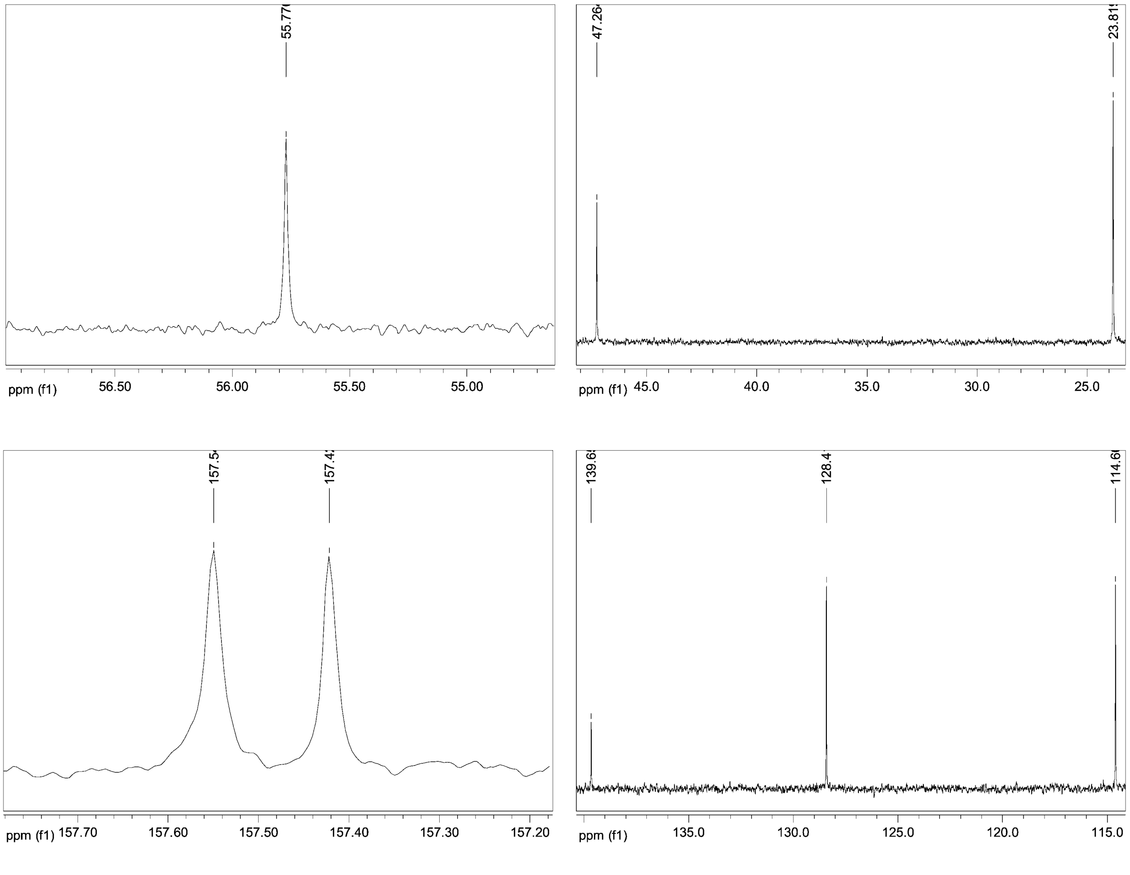

Figure S10: ESIMS *m*/*z* data for **4a**.

Acquisition Parameter:
Comment: 1/500 CH_2Cl_2 1/500 CH_3CN
Instrument: esquire6000

Ion Source Type	ESI	Ion Polarity	Positive	Alternating Ion Polarity	off
Mass Range Mode	Std/Normal	Scan Begin	65 *m/z*	Scan End	3000 *m/z*
Capillary Exit	143.5 Volt	Skim 1	40.0 Volt	Trap Drive	71.7
Accumulation Time	69093 µs	Averages	8 Spectra	Auto MS/MS	off

- ESIMS (+MS) *m*/*z*: 639.3 amu for [$C_{28}H_{46}Cl_2N_6O_2Pd$ (**4a**) − Cl^-]$^+$:

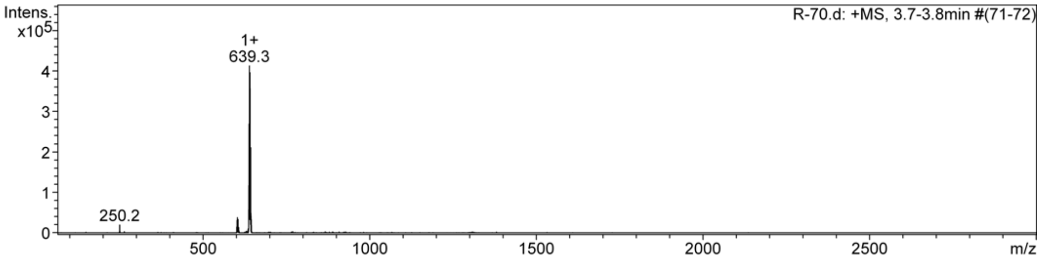

Magnification of the (+MS) *m*/*z*: 639.3 amu for [$C_{28}H_{46}Cl_2N_6O_2Pd$ (**4a**) − Cl^-]$^+$. Peak exhibited the isotopic distribution characteristic of the presence of Pd and Cl:

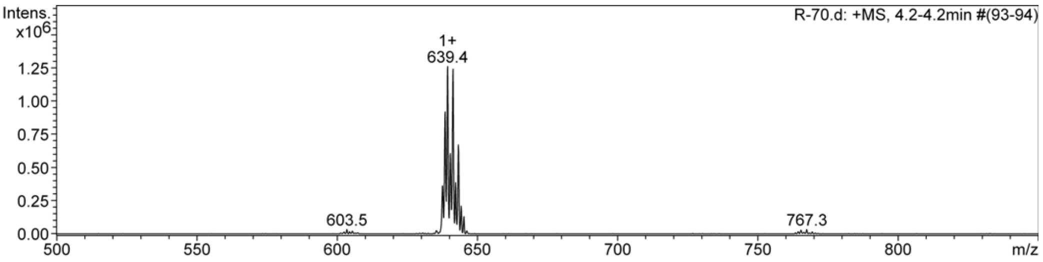

- ESIMS (−MS) *m*/*z*: 711.2 amu for [$C_{28}H_{46}Cl_2N_6O_2Pd$ (**4a**) + Cl^-]$^-$:

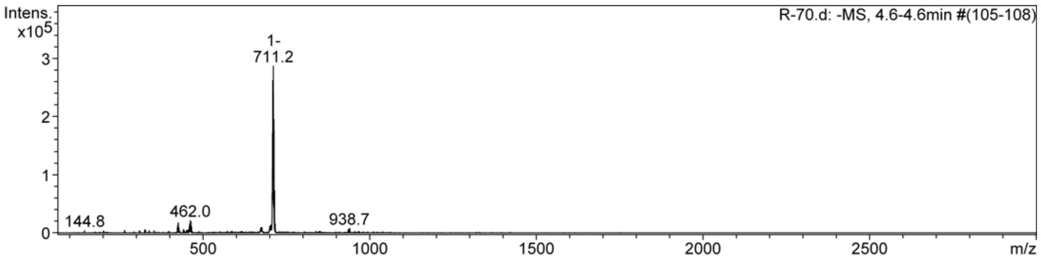

Magnification of the (−MS) *m*/*z*: 711.2 amu for [$C_{28}H_{46}Cl_2N_6O_2Pd$ (**4a**) + Cl^-]$^-$. Peak exhibited the isotopic distribution characteristic of the presence of Pd and Cl:

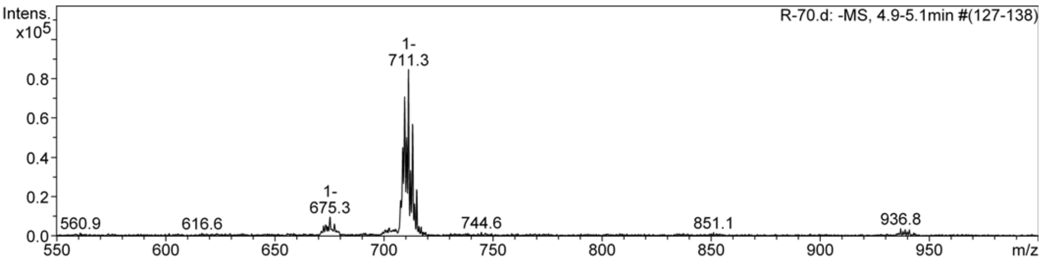

Table S2: Crystal data and structure refining for **4b**.

Empirical formula	$C_{28}H_{42}Cl_2N_6O_4Pd$	
Formula weight	703.98	
Temperature	173(2) K	
Wavelength	0.71073 Å	
Crystal system	Monoclinic	
Space group	P 2_1/n	
Unit cell dimensions	a = 11.2008(3) Å	α= 90°.
	b = 13.5909(4) Å	β= 111.5630(10)°.
	c = 11.5265(4) Å	γ = 90°.
Volume	1631.86(9) $Å^3$	
Z	2	
Density (calculated)	1.433 Mg/m^3	
Absorption coefficient	0.774 mm^{-1}	
F(000)	728	
Crystal size	0.25 x 0.20 x 0.15 mm^3	
Theta range for data collection	2.17 to 25.25°.	
Index ranges	-9<=h<=13, -16<=k<=16, -13<=l<=12	
Reflections collected	14535	
Independent reflections	2945 [R(int) = 0.0231]	
Completeness to theta = 25.25°	99.1%	
Absorption correction	Semi-empirical from equivalents	
Max. and min. transmission	0.8905 and 0.8301	
Refinement method	Full-matrix least-squares on F^2	
Data / restraints / parameters	2945 / 2 / 197	
Goodness-of-fit on F^2	1.081	
Final R indices [I>2sigma(I)]	R1 = 0.0261, wR2 = 0.0796	
R indices (all data)	R1 = 0.0307, wR2 = 0.0822	
Largest diff. peak and hole	0.404 and -0.203 e.$Å^{-3}$	

Figure S11: ORTEP drawing of **4b**.

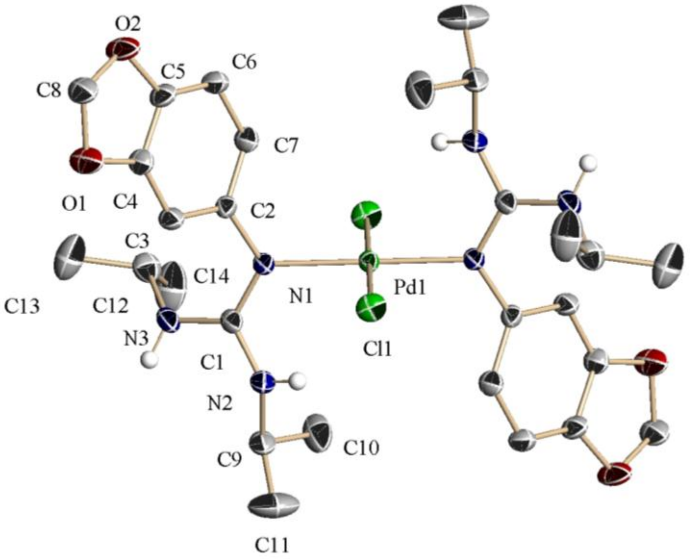

Crystal packing detail of **4b** viewed along the *a*-axis showing the presence of intermolecular hydrogen bonds between O (from the dioxole moieties) and H (NH groups) atoms (intramolecular hydrogen bonds are omitted for clarity):

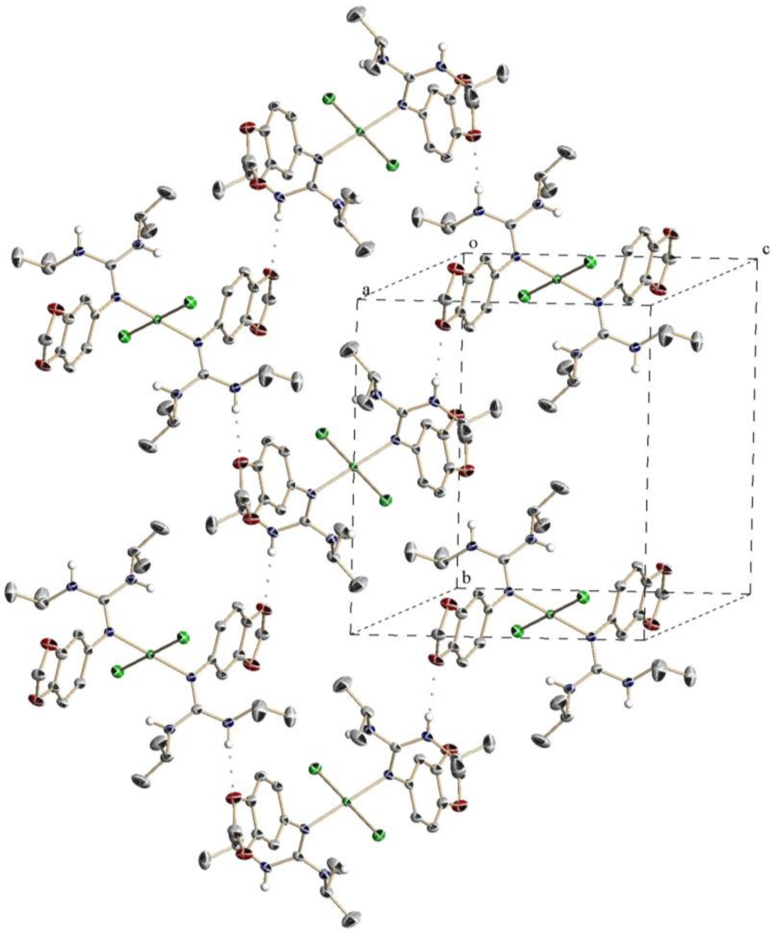

Figure S12: ^{1}H NMR spectrum in CD_2Cl_2 for isolated complex **4b**.

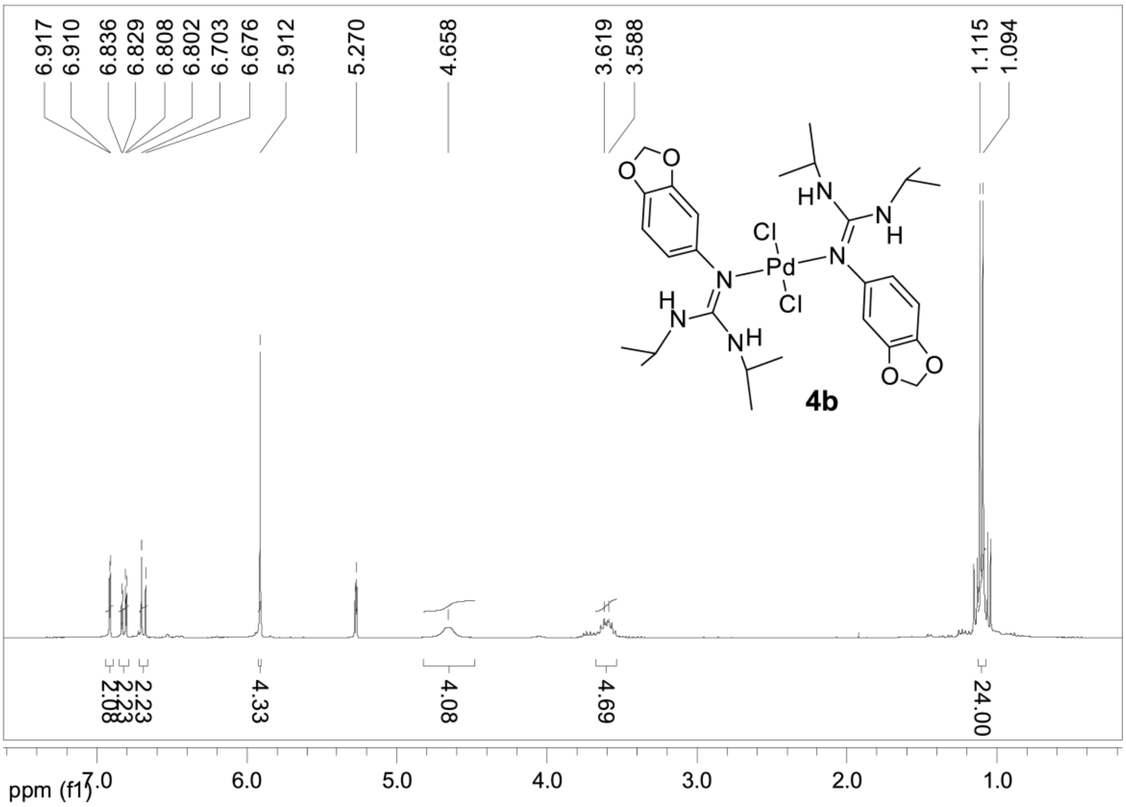

Magnification of the ^{1}H NMR peaks (**4b**):

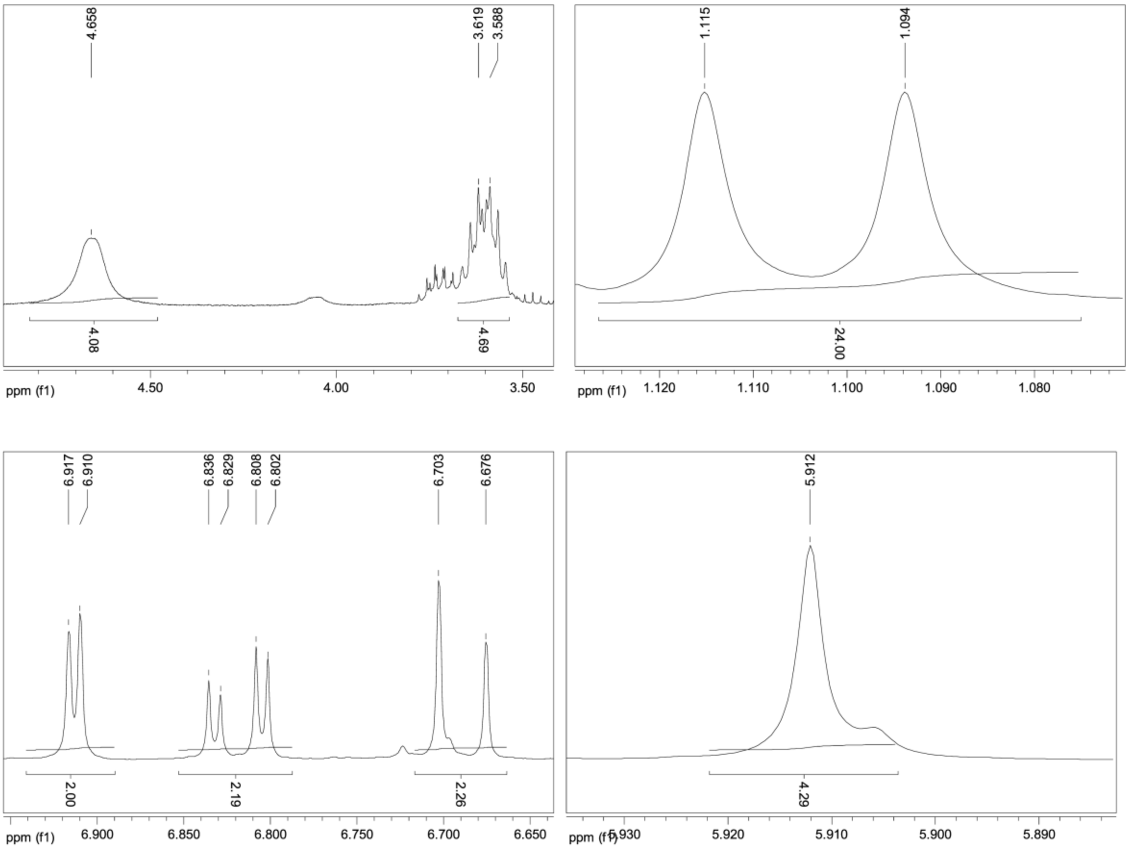

Figure S13: ^{13}C NMR spectrum in CD_2Cl_2 for isolated complex **4b**.

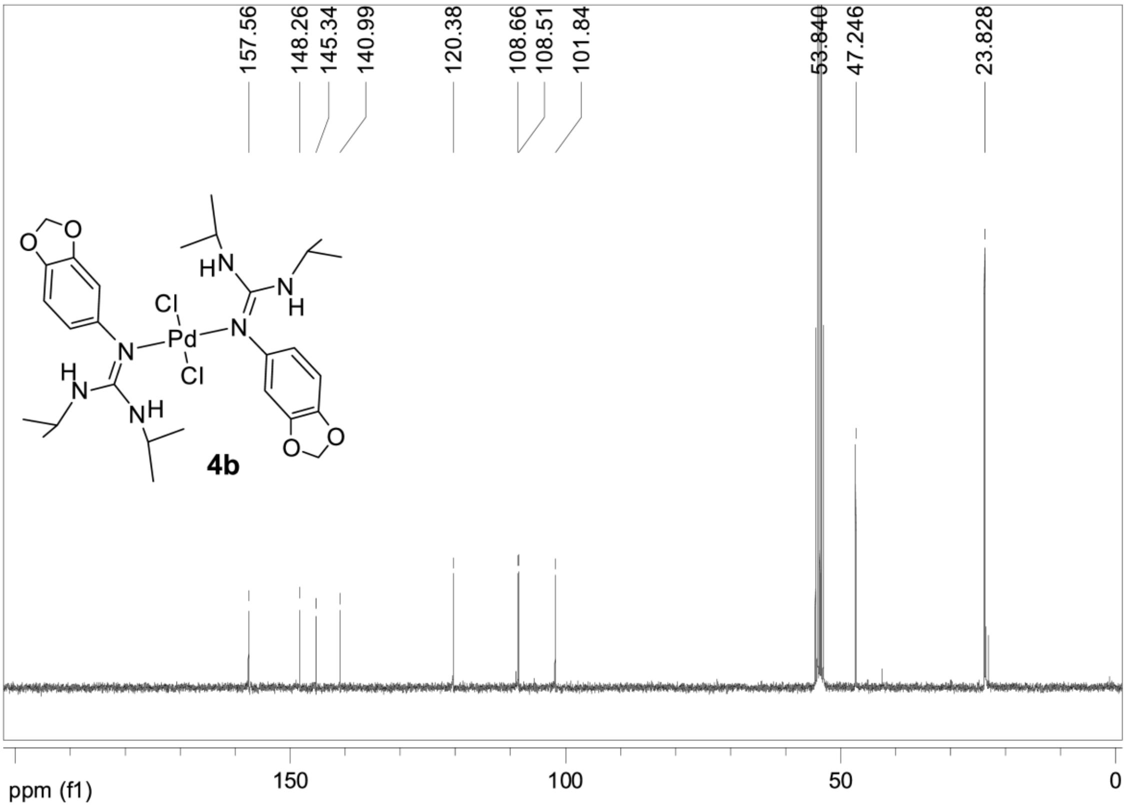

Magnification of the ^{13}C NMR peaks (**4b**).

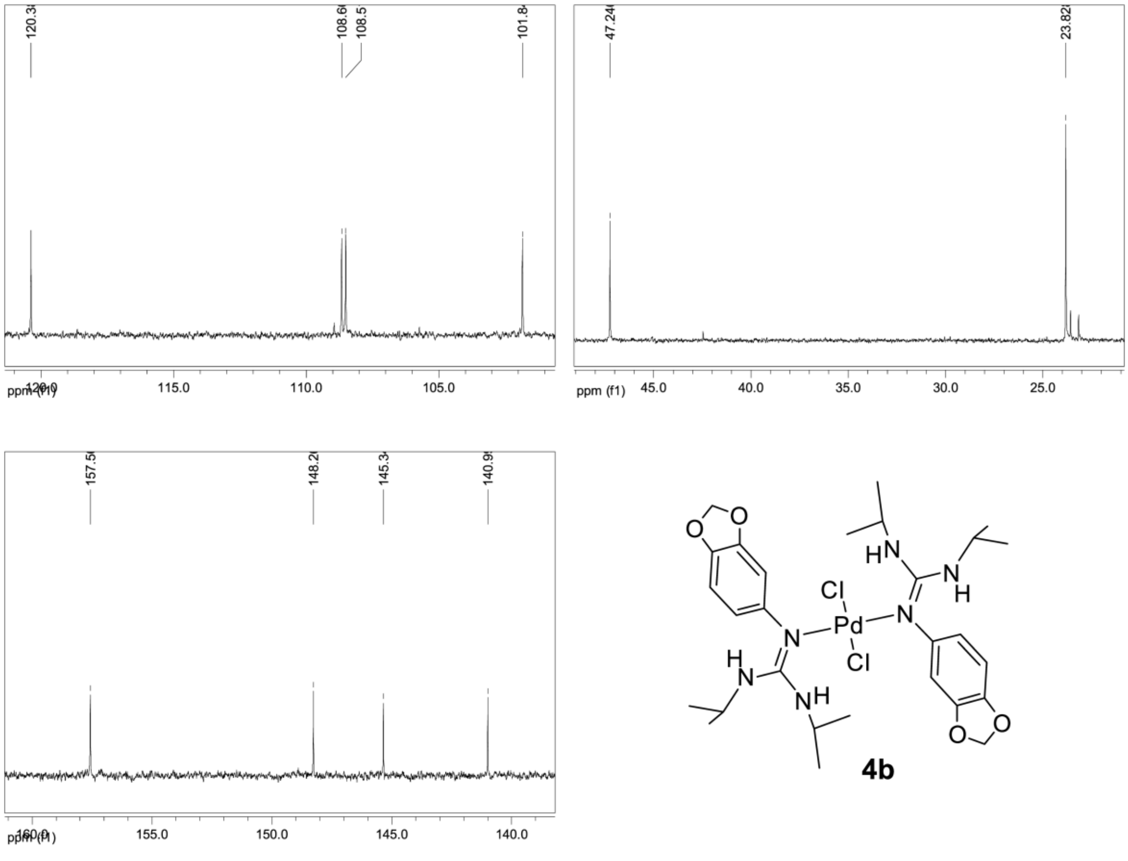

Figure S14: ESIMS *m/z* data for **4b**.

Acquisition Parameter:
Comment: 1/500 CH_2Cl_2 1/500 CH_3CN
Instrument: esquire6000

Ion Source Type	ESI	Ion Polarity	Positive	Alternating Ion Polarity	off		
Mass Range Mode	Std/Normal	Scan Begin	65 *m/z*	Scan End	3000 *m/z*		
Capillary Exit	143.5 Volt	Skim 1	40.0 Volt	Trap Drive	71.7		
Accumulation Time	42115 µs	Averages	8 Spectra	Auto MS/MS	off		

- ESIMS (+MS) *m/z*: 667.2 amu for [$C_{28}H_{42}Cl_2N_6O_4Pd$ (**4b**) − Cl^-]$^+$:

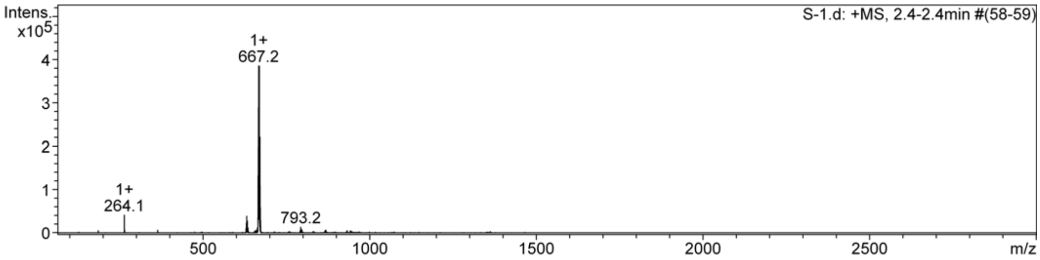

Magnification of the (+MS) *m/z*: 667.2 amu for [$C_{28}H_{42}Cl_2N_6O_4Pd$ (**4b**) − Cl−]$^+$. Peak exhibited the isotopic distribution characteristic of the presence of Pd and Cl:

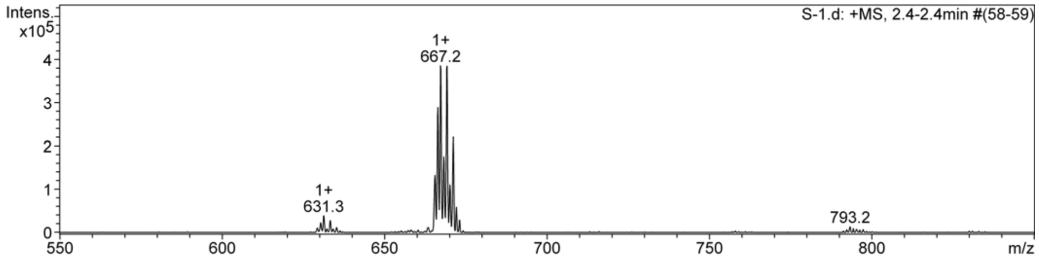

- ESIMS (−MS) *m/z*: 739.1 amu for [$C_{28}H_{42}Cl_2N_6O_4Pd$ (**4b**) + Cl^-]$^-$:

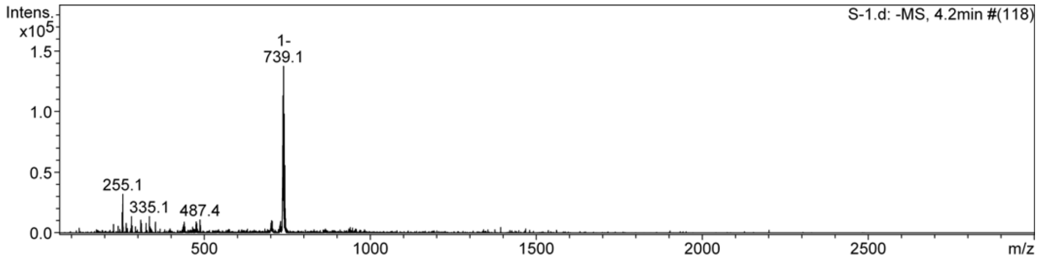

Magnification of the (−MS) *m/z*: 739.1 amu for [$C_{28}H_{42}Cl_2N_6O_4Pd$ (**4b**) + Cl^-]$^-$. Peak exhibited the isotopic distribution characteristic of the presence of Pd and Cl:

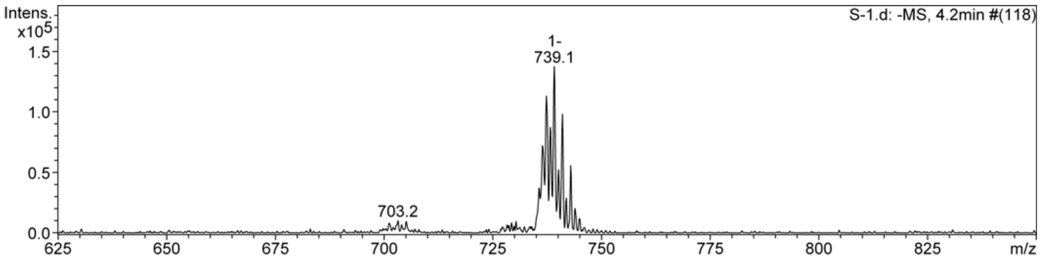

Table S3: Crystal data and structure refining for **4c**.

Empirical formula	$C_{26}H_{38}Cl_2F_2I_2N_6Pd$	
Formula weight	903.72	
Temperature	173(2) K	
Wavelength	0.71073 Å	
Crystal system	Monoclinic	
Space group	P 2_1/c	
Unit cell dimensions	a = 24.0900(11) Å	α= 90°.
	b = 11.4512(5) Å	β= 114.6710(10)°.
	c = 20.3122(8) Å	γ = 90°.
Volume	5091.8(4) $Å^3$	
Z	6	
Density (calculated)	1.768 Mg/m^3	
Absorption coefficient	2.559 mm^{-1}	
F(000)	2640	
Crystal size	0.10 x 0.10 x 0.08 mm^3	
Theta range for data collection	2.01 to 25.25°.	
Index ranges	-28<=h<=28, -13<=k<=13, -20<=l<=24	
Reflections collected	53653	
Independent reflections	9183 [R(int) = 0.0636]	
Completeness to theta = 25.25°	99.8%	
Absorption correction	Semi-empirical from equivalents	
Max. and min. transmission	0.8154 and 0.7819	
Refinement method	Full-matrix least-squares on F^2	
Data / restraints / parameters	9183 / 6 / 556	
Goodness-of-fit on F^2	1.071	
Final R indices [I>2sigma(I)]	R1 = 0.0452, wR2 = 0.1072	
R indices (all data)	R1 = 0.0675, wR2 = 0.1160	
Largest diff. peak and hole	1.902 and -1.513 e.$Å^{-3}$	

Figure S15: ORTEP drawing of **4c**.

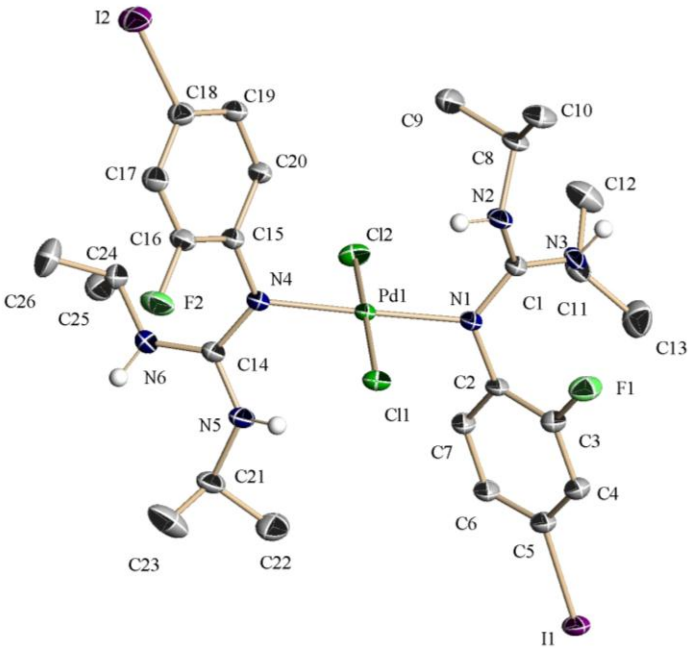

Crystal packing detail of **4c** viewed along the *a*-axis showing the presence of intermolecular hydrogen bonds between Cl and H (NH groups) atoms (intramolecular hydrogen bonds are omitted for clarity):

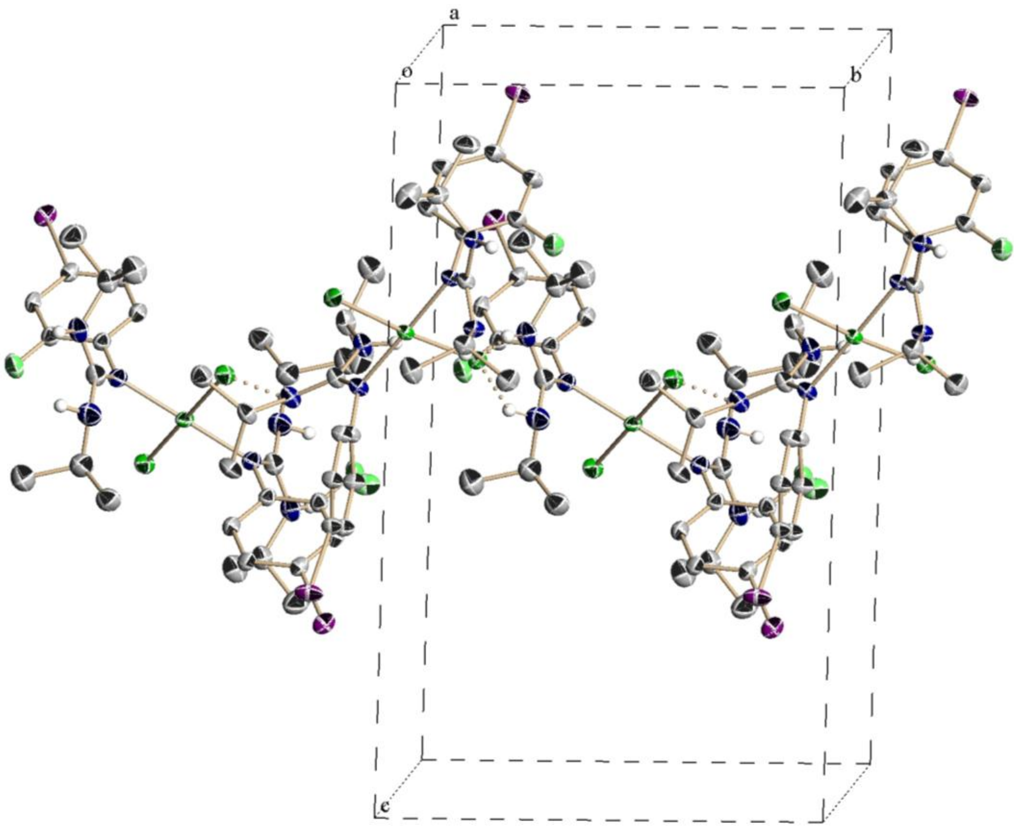

Figure S16: ^{1}H NMR spectrum in CD_2Cl_2 for isolated complex **4c**.

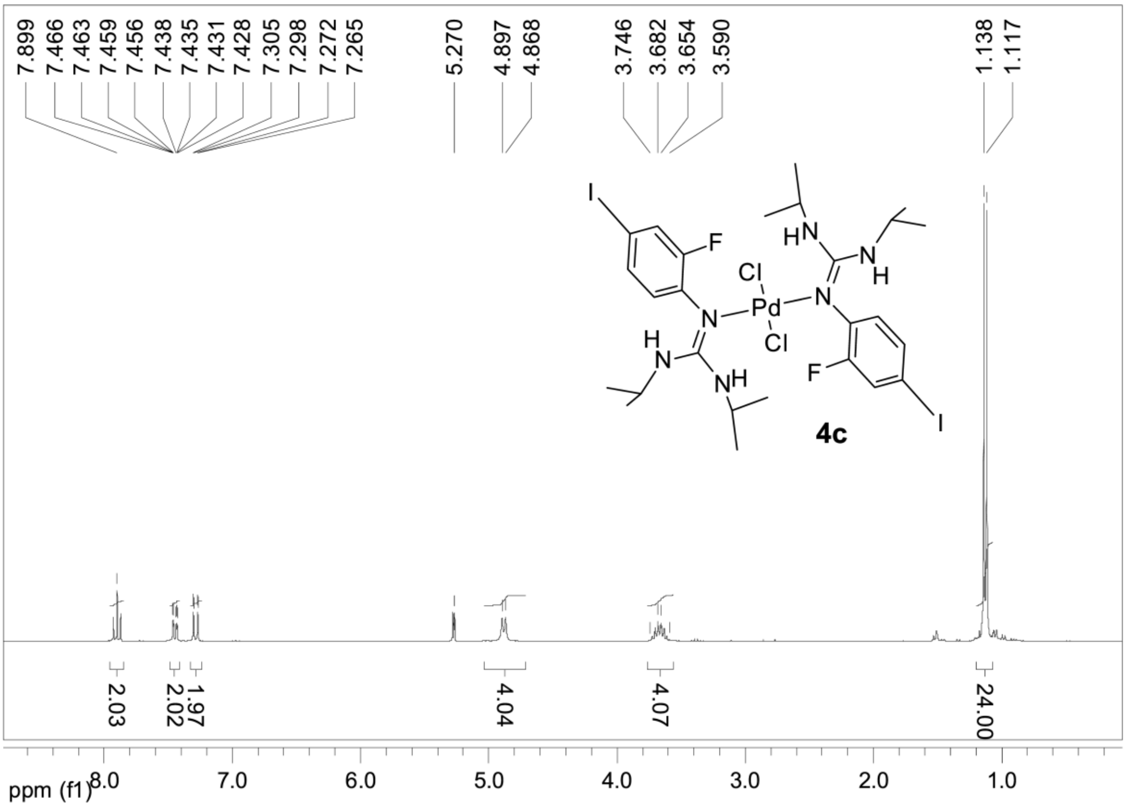

Magnification of the ^{1}H NMR peaks (**4c**).

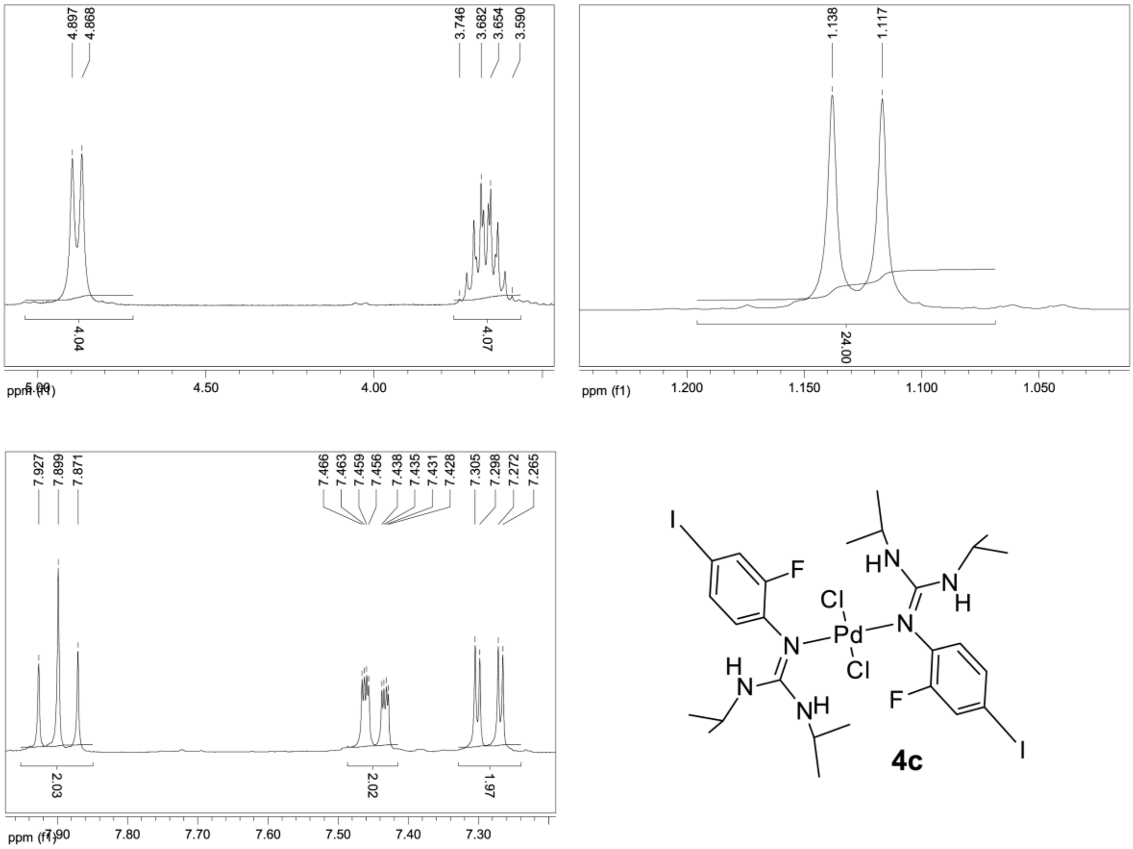

Figure S17: ^{19}F NMR spectrum in CD_2Cl_2 for isolated complex **4c**.

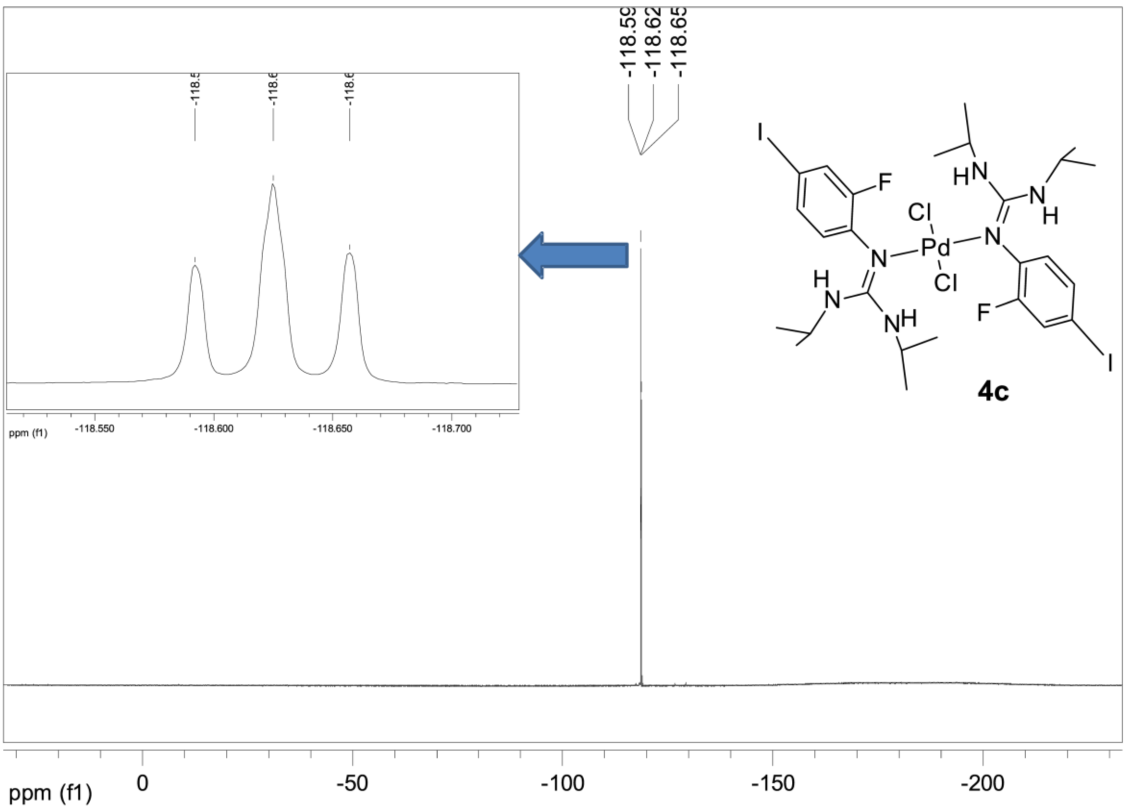

Figure S18: ^{13}C NMR spectrum in CD_2Cl_2 for isolated complex **4c**.

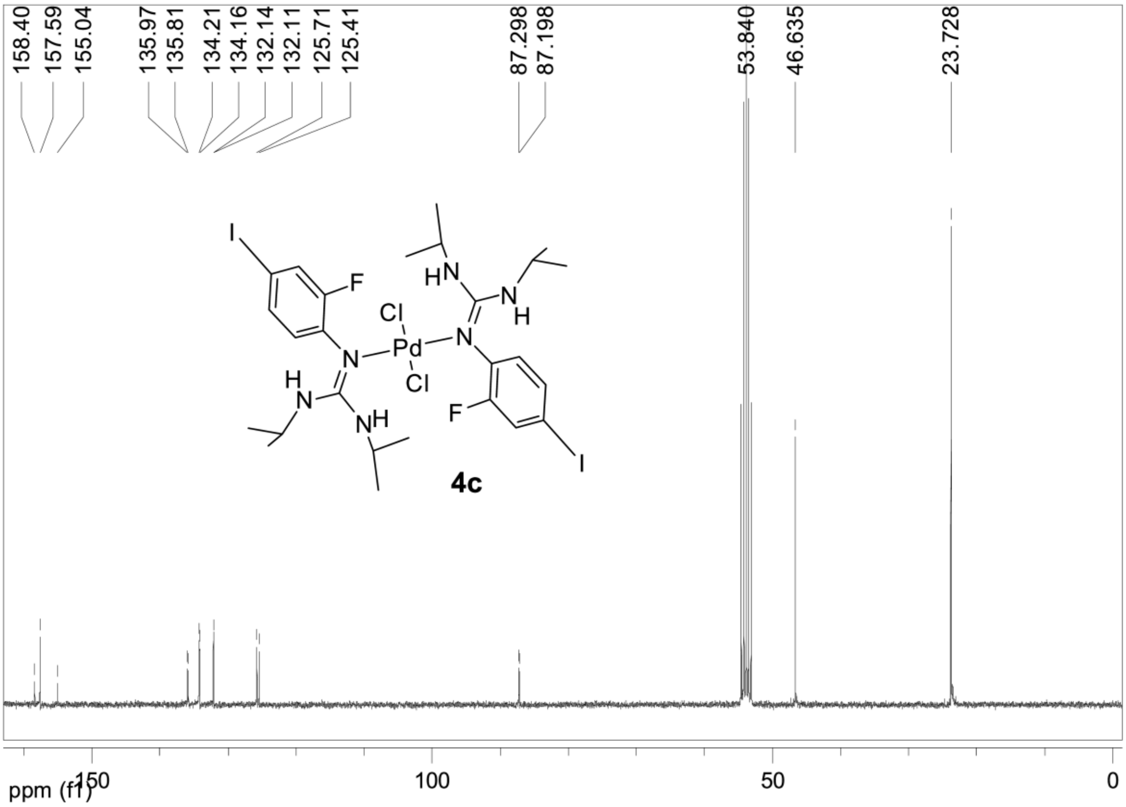

Magnification of the ^{13}C NMR peaks (**4c**).

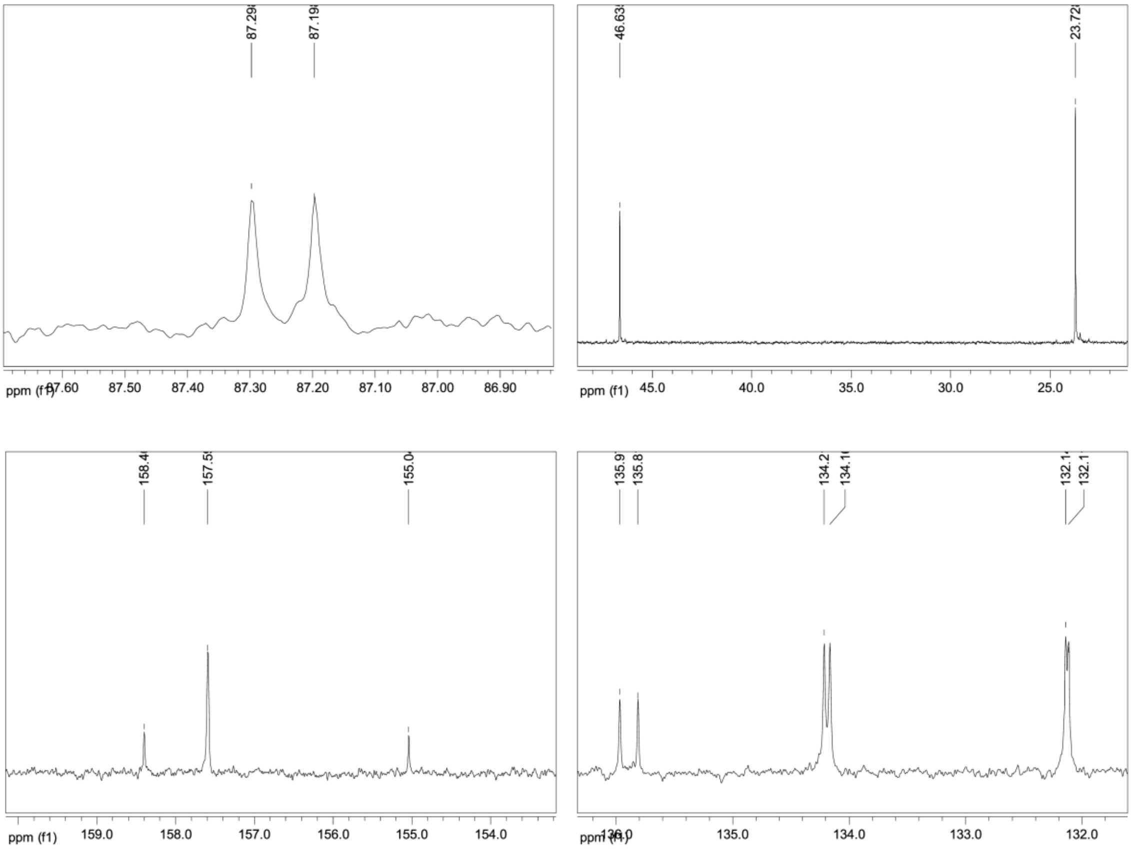

Figure S19: ESIMS *m*/*z* data for **4c**.

Acquisition Parameter:
Comment: 1/500 CH_2Cl_2 1/500 CH_3CN
Instrument: esquire6000

Ion Source Type	ESI	Ion Polarity	Positive	Alternating Ion Polarity	off		
Mass Range Mode	Std/Normal	Scan Begin	65 *m/z*	Scan End	1500 *m/z*		
Capillary Exit	158.5 Volt	Skim 1	40.0 Volt	Trap Drive	86.3		
Accumulation Time	30617 µs	Averages	8 Spectra	Auto MS/MS	off		

- ESIMS (+MS) *m*/*z*: 867.1 amu for [$C_{26}H_{38}Cl_2F_2I_2N_6Pd$ (**4c**) – Cl^-]$^+$:

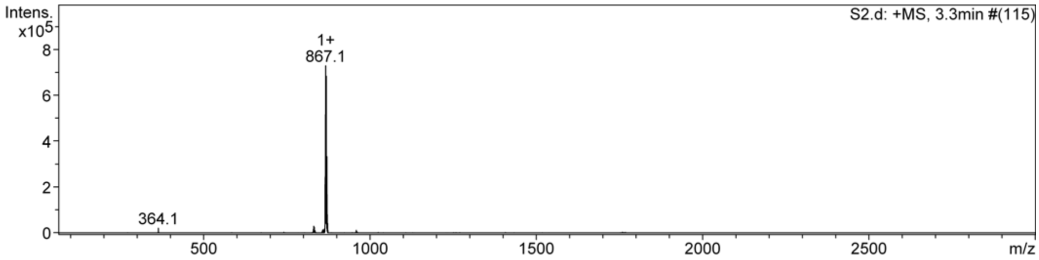

Magnification of the (+MS) *m*/*z*: 867.1 amu for [$C_{26}H_{38}Cl_2F_2I_2N_6Pd$ (**4c**) − Cl^-]$^+$. Peak exhibited the isotopic distribution characteristic of the presence of Pd and Cl:

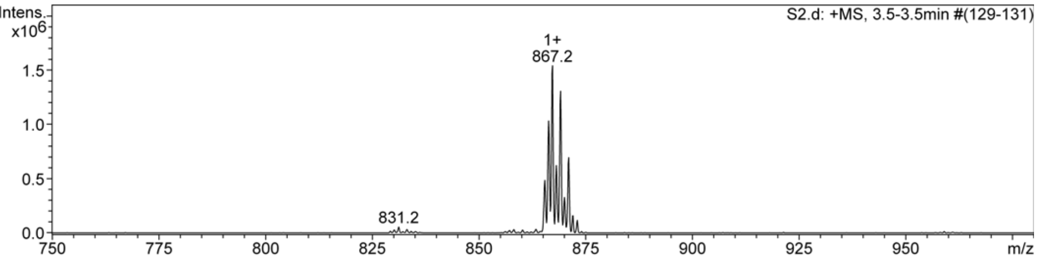

- ESIMS (−MS) *m*/*z*: 938.9 amu for [$C_{26}H_{38}Cl_2F_2I_2N_6Pd$ (**4c**) + Cl^-]$^-$:

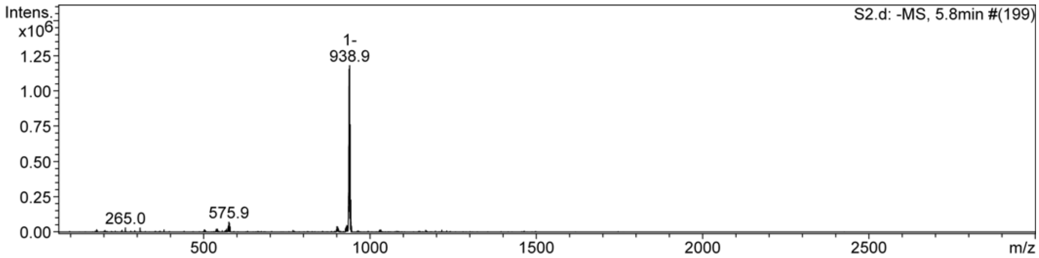

Magnification of the (−MS) *m*/*z*: 938.9 amu for [$C_{26}H_{38}Cl_2F_2I_2N_6Pd$ (**4c**) + Cl^-]$^-$. Peak exhibited the isotopic distribution characteristic of the presence of Pd and Cl:

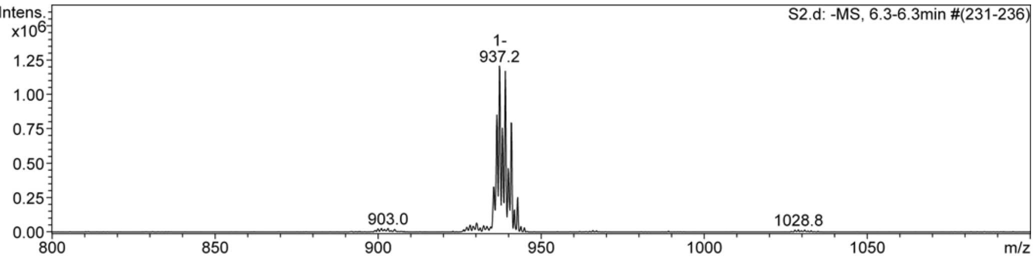

Table S4: Crystal data and structure refining for **5a**.

Empirical formula	$C_{14}H_{23}N_3O$	
Formula weight	249.35	
Temperature	173(2) K	
Wavelength	0.71073 Å	
Crystal system	Monoclinic	
Space group	C c	
Unit cell dimensions	a = 12.900(7) Å	α= 90°.
	b = 13.630(7) Å	β= 90.176(16)°.
	c = 8.531(4) Å	γ = 90°.
Volume	1500.0(13) $Å^3$	
Z	4	
Density (calculated)	1.104 Mg/m^3	
Absorption coefficient	0.071 mm^{-1}	
F(000)	544	
Crystal size	0.35 x 0.10 x 0.10 mm^3	
Theta range for data collection	2.17 to 25.27°.	
Index ranges	-15<=h<=15, -16<=k<=16, -10<=l<=10	
Reflections collected	6674	
Independent reflections	1371 [R(int) = 0.0947]	
Completeness to theta = 25.27°	99.6%	
Absorption correction	Semi-empirical from equivalents	
Max. and min. transmission	0.9929 and 0.9755	
Refinement method	Full-matrix least-squares on F^2	
Data / restraints / parameters	1371 / 0 / 169	
Goodness-of-fit on F^2	1.015	
Final R indices [I>2sigma(I)]	R1 = 0.0681, wR2 = 0.1352	
R indices (all data)	R1 = 0.0805, wR2 = 0.1870	
Largest diff. peak and hole	0.333 and -0.308 e.$Å^{-3}$	

Figure S20: ORTEP drawing of **5a**.

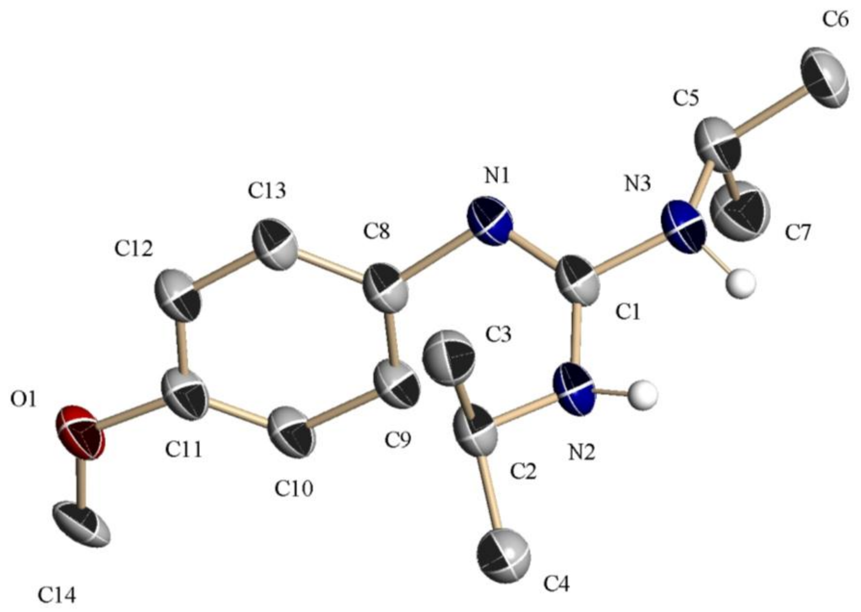

Crystal packing detail of **5a** viewed along the *b*-axis showing the presence of intermolecular hydrogen bonds between N atoms.

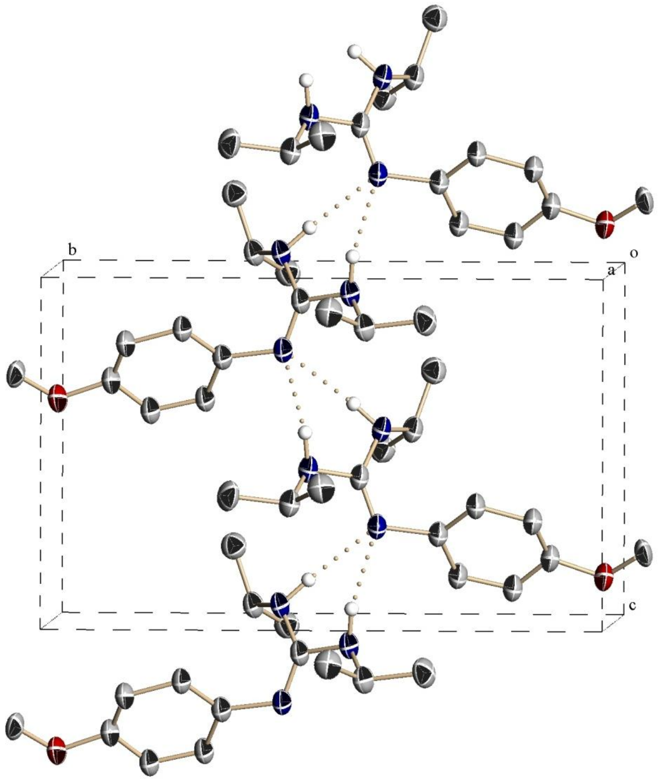

Figure S21: ^{1}H NMR spectrum in CD_2Cl_2 for isolated complex **5a**.

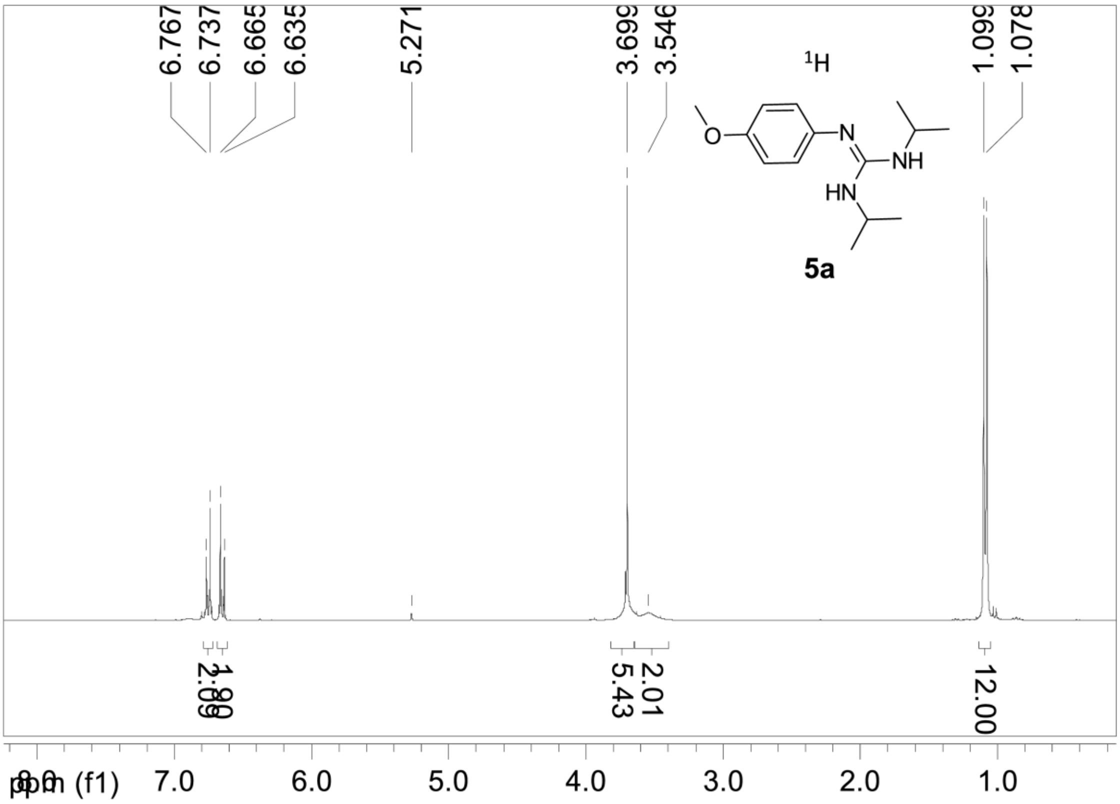

Magnification of some ^{1}H NMR peaks (**5a**).

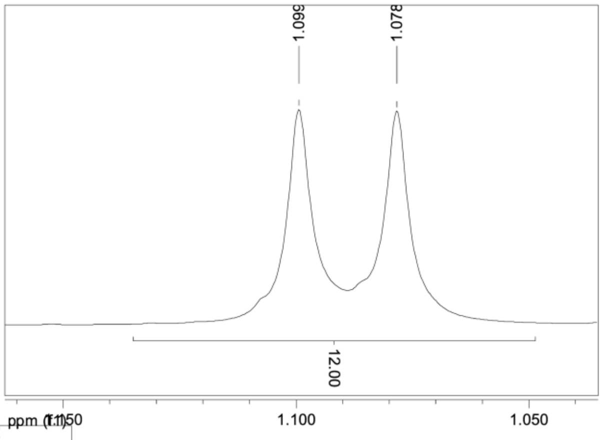

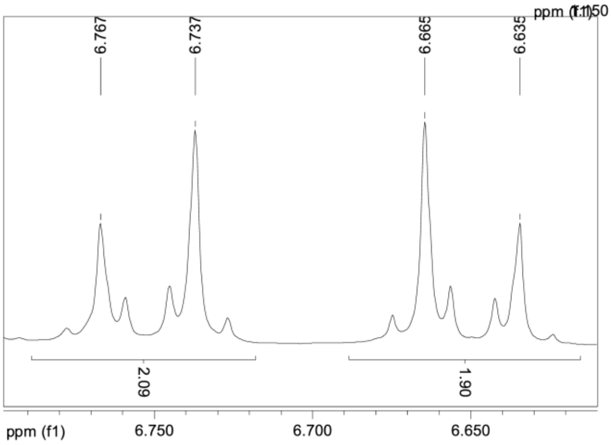

Figure S22: ^{13}C NMR spectrum in CD_2Cl_2 for isolated complex **5a**.

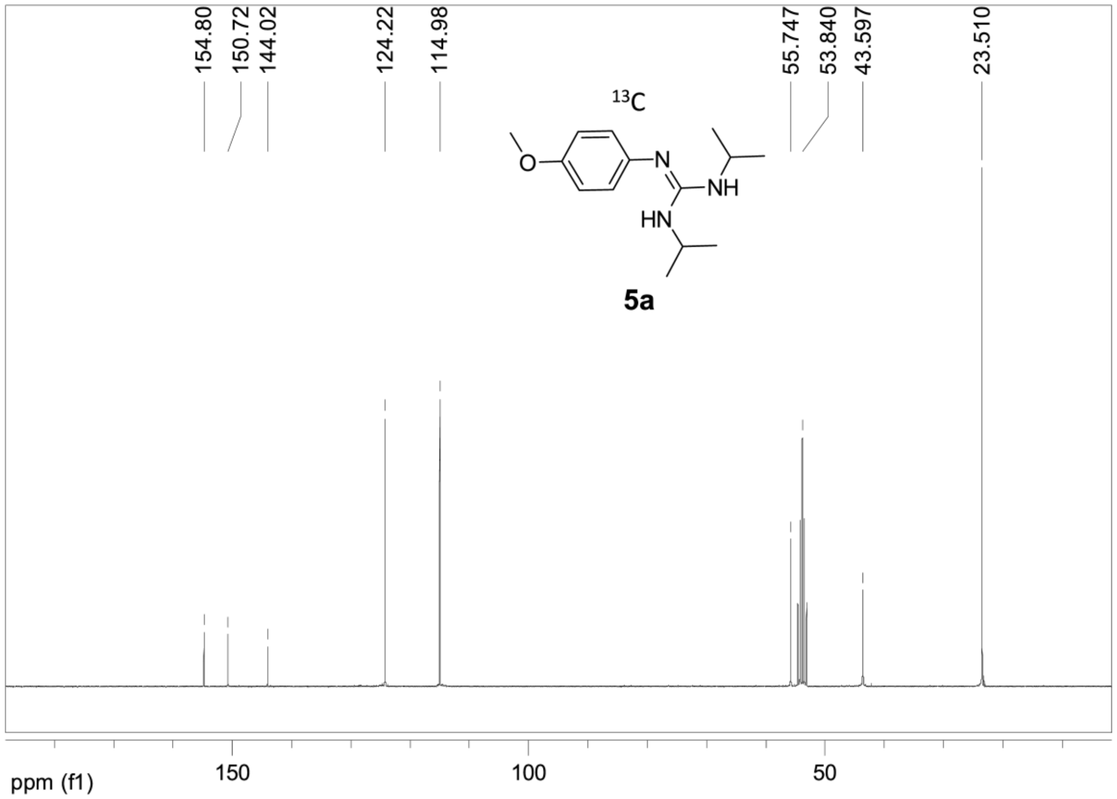

Magnification of ^{13}C NMR peaks (**5a**).

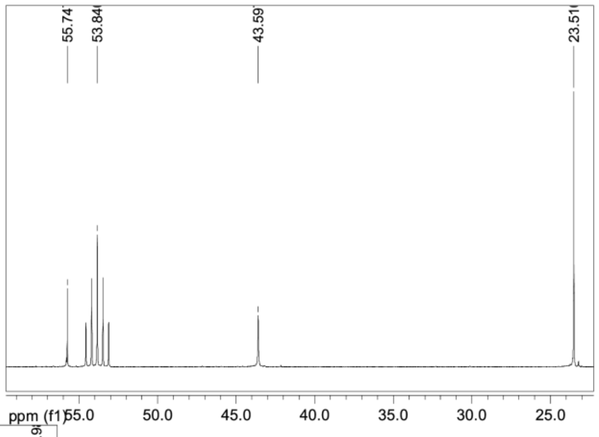

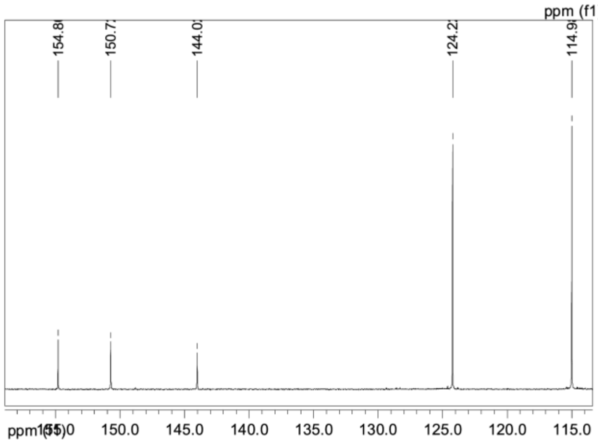

Figure S23: ESIMS *m*/*z* data for **5a**.

Acquisition Parameter:
Comment: 1/1000 CH_3CN 1/1000 MeOH
Instrument: esquire6000

Ion Source Type	ESI	Ion Polarity	Positive	Alternating Ion Polarity	off		
Mass Range Mode	Std/Normal	Scan Begin	65 *m*/*z*	Scan End	600 *m*/*z*		
Capillary Exit	109.7 Volt	Skim 1	40.0 Volt	Trap Drive	52.9		
Accumulation Time	594 µs	Averages	8 Spectra	Auto MS/MS	off		

ESIMS (+MS) *m*/*z*: 250.2 amu for $[C_{14}H_{23}N_3O$ (**5a**) $+ H^+]^+$:

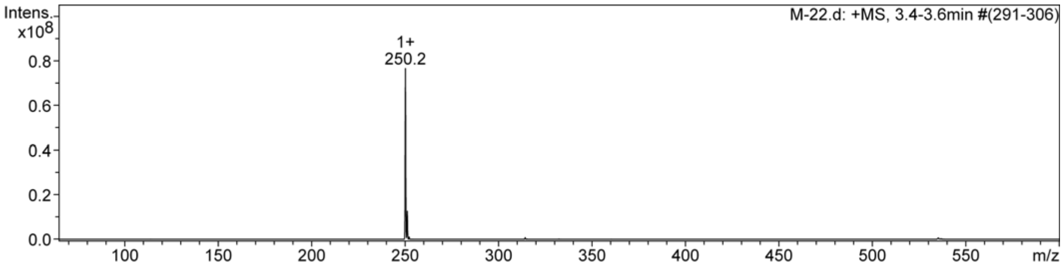

MS corresponding to the fragmentation of the peak (250.2) marked with diamond:

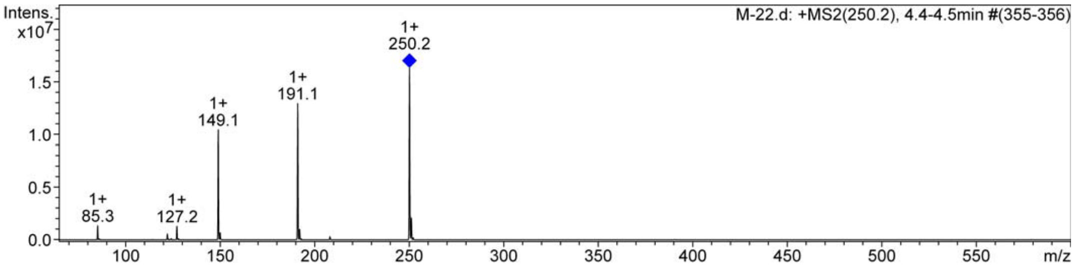

Figure S24: ^{1}H NMR spectrum in CD_2Cl_2 for isolated complex **5b**.

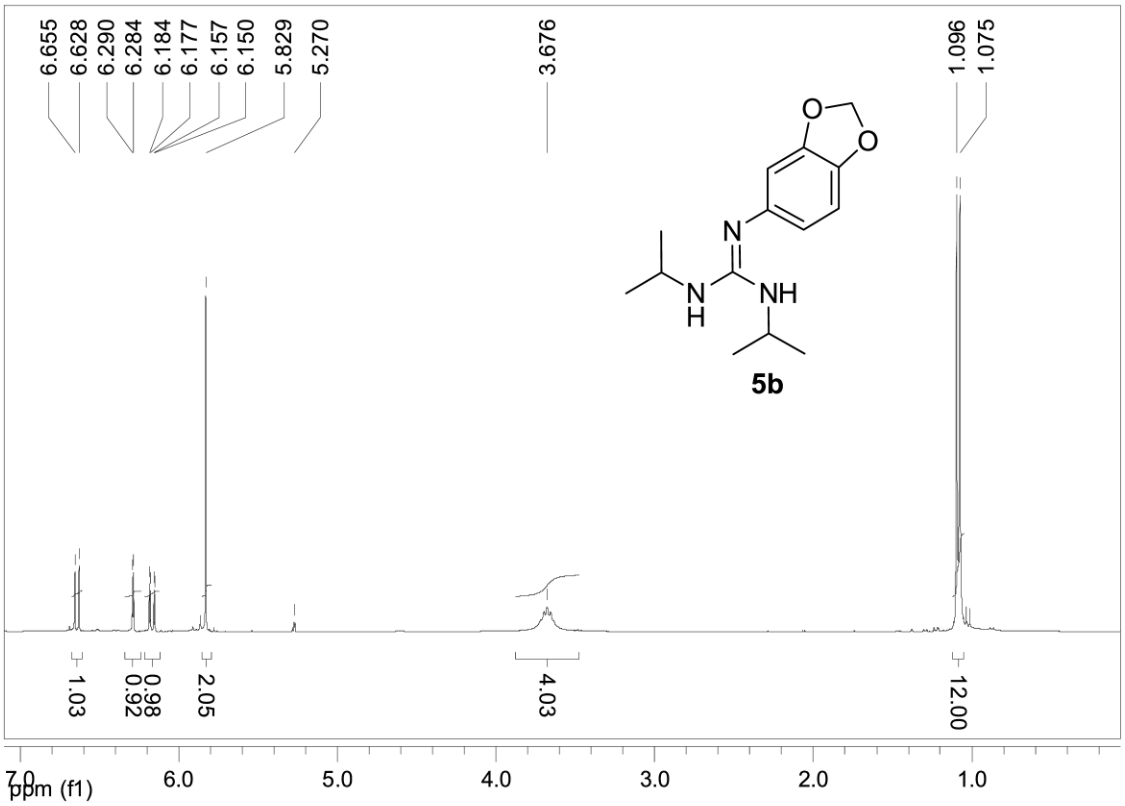

Magnification of ^{1}H NMR peaks (**5b**).

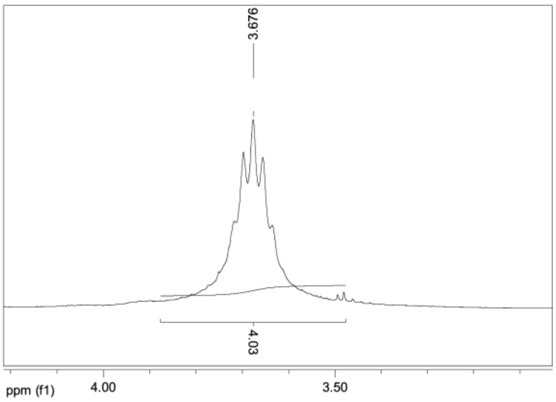

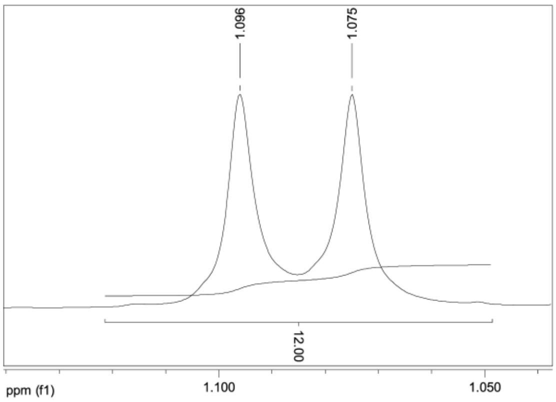

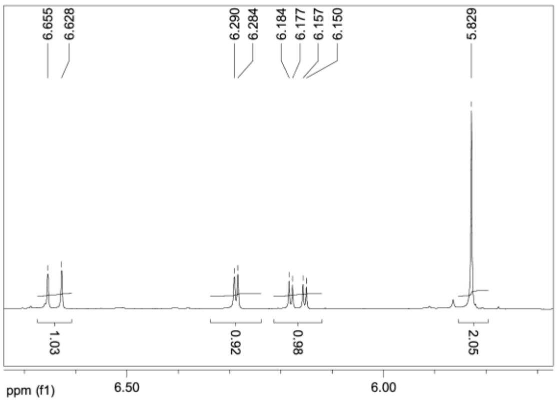

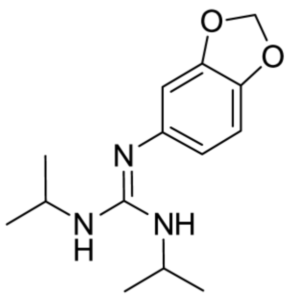

5b

Figure S25: ^{13}C NMR spectrum in CD_2Cl_2 for isolated complex **5b**.

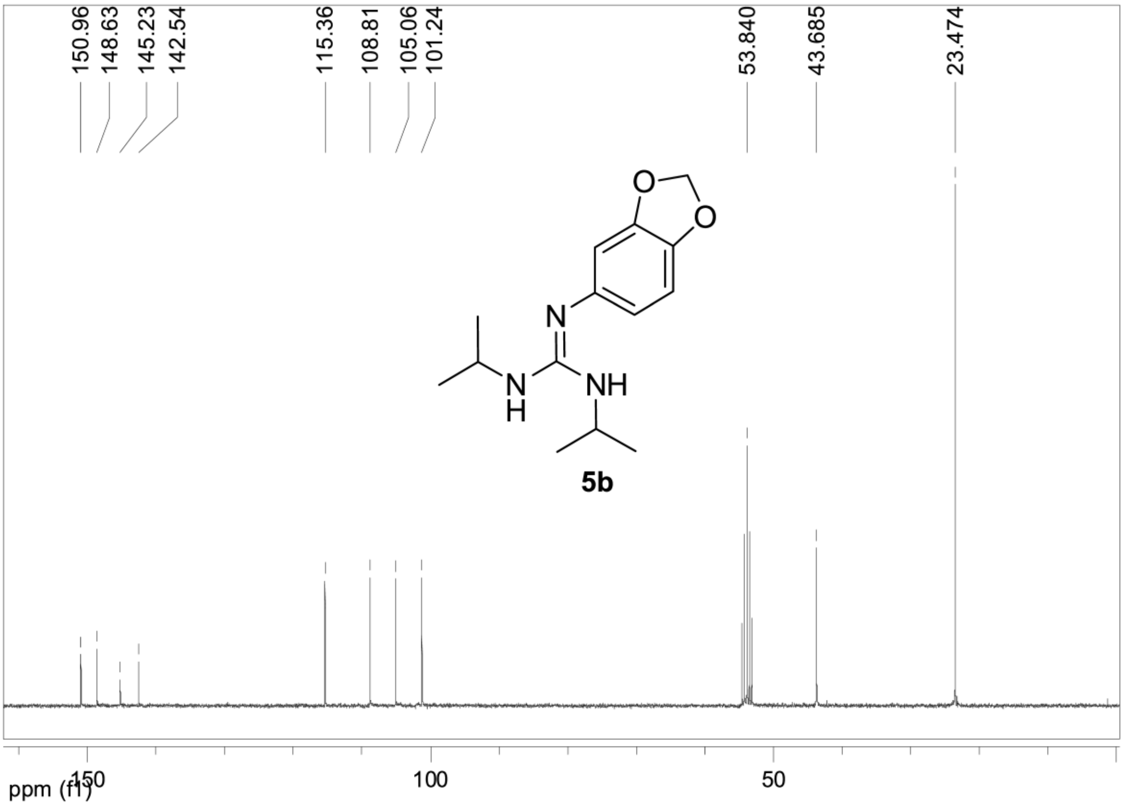

Magnification of ^{13}C NMR peaks (**5b**).

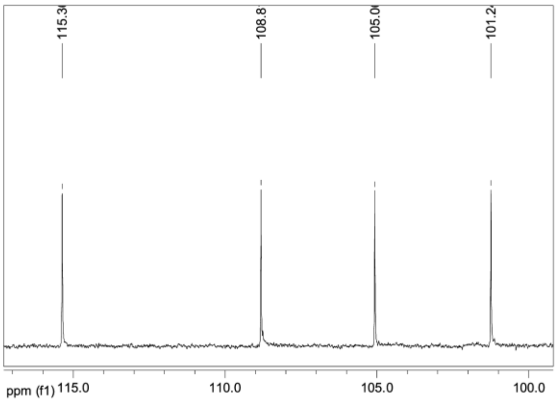

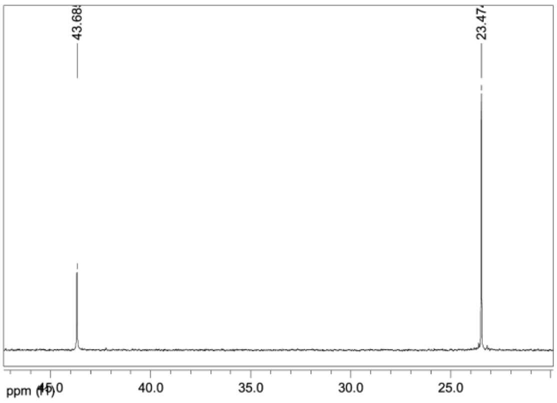

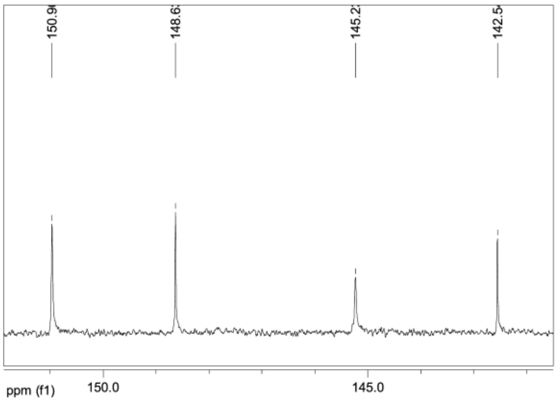

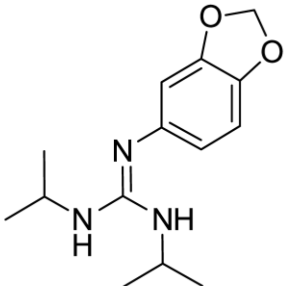

5b

Figure S26: GC–MS *m/z* data for **5b**.

Compound [$C_{14}H_{21}N_3O_2$ (**5b**)] (263.2).

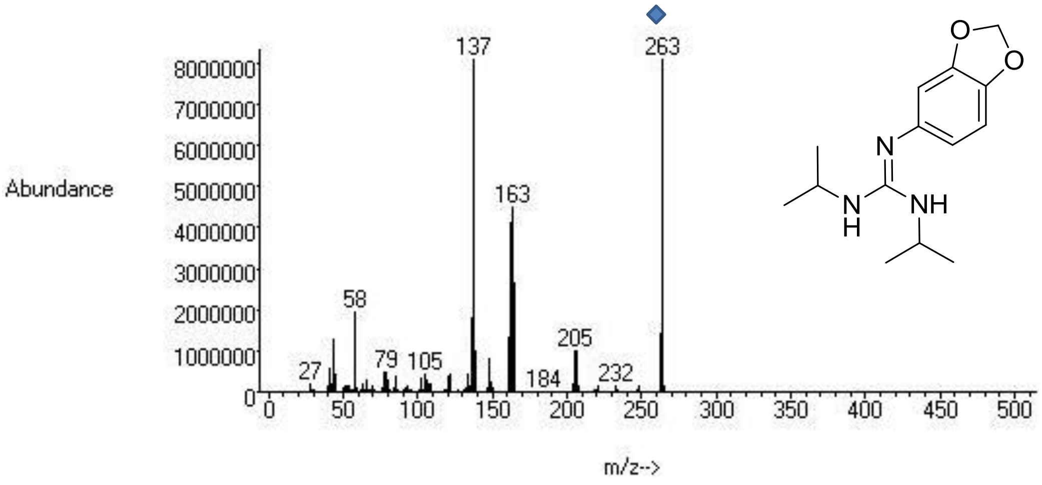

MS corresponding to the magnification of the peak (263.2) marked with a diamond.

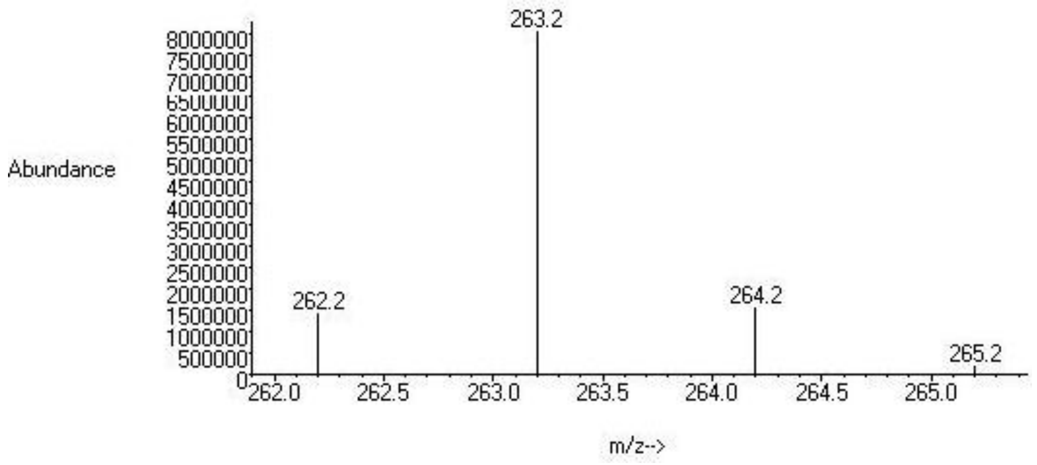

Supplement: File 1 — Experimental details of preparation, isolation and full characterization of new palladium compounds 3a–c, 4a–c as well as guanidine compounds 5a,b, including IR, NMR, ESIMS and GC–MS spectra for new compounds. [file Beilstein_J_Org_Chem-09-1455-s001.pdf]
